# Predicting the spatial distribution of wintering golden eagles to inform full annual cycle conservation in western North America

**DOI:** 10.1371/journal.pone.0297345

**Published:** 2024-01-31

**Authors:** Zachary P. Wallace, Bryan E. Bedrosian, Jeffrey R. Dunk, David W. LaPlante, Brian Woodbridge, Brian W. Smith, Jessi L. Brown, Todd M. Lickfett, Katherine Gura, Dave Bittner, Ross H. Crandall, Rob Domenech, Todd E. Katzner, Kevin J. Kritz, Stephen B. Lewis, Michael J. Lockhart, Tricia A. Miller, Katie Quint, Adam Shreading, Steve J. Slater, Dale W. Stahlecker

**Affiliations:** 1 Wyoming Natural Diversity Database, University of Wyoming, Laramie, Wyoming, United States of America; 2 Teton Raptor Center, Wilson, Wyoming, United States of America; 3 Department of Environmental Science and Management, California State Polytechnic University, Humboldt, Arcata, California, United States of America; 4 Natural Resource Geospatial, Yreka, California, United States of America; 5 California State Polytechnic University, Humboldt, Arcata, California, United States of America; 6 U.S. Fish and Wildlife Service, Denver, Colorado, United States of America; 7 Sparrowhawk Data Science, Reno, Nevada, United States of America; 8 Wildlife Research Institute, Inc., Julian, California, United States of America; 9 Wyoming Game and Fish Department, Habitat Protection Program, Pinedale, Wyoming, United States of America; 10 Raptor View Research Institute, Missoula, Montana, United States of America; 11 U.S. Geological Survey, Boise, Idaho, United States of America; 12 U.S. Fish and Wildlife Service, Juneau, Alaska, United States of America; 13 Wildlands Bio-Consulting, Laramie, Wyoming, United States of America; 14 Conservation Science Global, Cape May, New Jersey, United States of America; 15 HawkWatch, International, Salt Lake City, Utah, United States of America; 16 Eagle Environmental, Inc., Santa Fe, New Mexico, United States of America; San Diego Zoo Institute for Conservation Research, UNITED STATES

## Abstract

Wildlife conservation strategies focused on one season or population segment may fail to adequately protect populations, especially when a species’ habitat preferences vary among seasons, age-classes, geographic regions, or other factors. Conservation of golden eagles (*Aquila chrysaetos*) is an example of such a complex scenario, in which the distribution, habitat use, and migratory strategies of this species of conservation concern vary by age-class, reproductive status, region, and season. Nonetheless, research aimed at mapping priority use areas to inform management of golden eagles in western North America has typically focused on territory-holding adults during the breeding period, largely to the exclusion of other seasons and life-history groups. To support population-wide conservation planning across the full annual cycle for golden eagles, we developed a distribution model for individuals in a season not typically evaluated–winter–and in an area of the interior western U.S. that is a high priority for conservation of the species. We used a large GPS-telemetry dataset and library of environmental variables to develop a machine-learning model to predict spatial variation in the relative intensity of use by golden eagles during winter in Wyoming, USA, and surrounding ecoregions. Based on a rigorous series of evaluations including cross-validation, withheld and independent data, our winter-season model accurately predicted spatial variation in intensity of use by multiple age- and life-history groups of eagles not associated with nesting territories (i.e., all age classes of long-distance migrants, and resident non-adults and adult “floaters”, and movements of adult territory holders and their offspring outside their breeding territories). Important predictors in the model were wind and uplift (40.2% contribution), vegetation and landcover (27.9%), topography (14%), climate and weather (9.4%), and ecoregion (8.7%). Predicted areas of high-use winter habitat had relatively low spatial overlap with nesting habitat, suggesting a conservation strategy targeting high-use areas for one season would capture as much as half and as little as one quarter of high-use areas for the other season. The majority of predicted high-use habitat (top 10% quantile) occurred on private lands (55%); lands managed by states and the Bureau of Land Management (BLM) had a lower amount (33%), but higher concentration of high-use habitat than expected for their area (1.5–1.6x). These results will enable those involved in conservation and management of golden eagles in our study region to incorporate spatial prioritization of wintering habitat into their existing regulatory processes, land-use planning tasks, and conservation actions.

## Introduction

Strategies to conserve wildlife populations are most effective when they capture the full range of habitats and behaviors within a species’ lifecycle [1]. Conservation planning can be especially complex for species with habitat preferences and migratory strategies that vary among seasons, age-classes, regional sub-populations, and other factors. For such species, management strategies focused on one season or group may fail to adequately protect the larger population. For example, conservation efforts on breeding grounds have failed to slow declines in populations of some grassland birds because those populations are primarily influenced by loss of wintering habitat [2]. Similarly, a network of conservation areas based on breeding habitat of greater sage-grouse (*Centrocercus urophasianus*) protected only 65% of winter locations [3]. Conservation planning is further complicated when such differences are driven by multiple factors, as was the case for moose (*Alces alces*) that exhibited variation in habitat associations and migratory strategies among both age-classes and geographic regions [4].

Management of golden eagles (*Aquila chrysaetos*) is an example of such a complex scenario, in which the distribution, habitat use, and migratory strategies of this species varies by age-class, reproductive status, region, and season [5]. Regional conservation planning for golden eagles will therefore be most effective if it accounts for both seasonal variation in the composition of the population in a given area and potential differences in the breeding status-, age-, and sex-specific habitat components used by eagles that occur there. For example, golden eagle populations in the western U.S. during winter are a mixture of year-round resident adult territory-holders, long-distance migrants from Alaska and northern Canada, short-distance migrants from neighboring regions, non-breeding adult resident “floaters”, and pre-breeding aged individuals from both resident and migrant populations [6]. Despite this complexity, research aimed at mapping priority use areas to inform management of golden eagles in western North America has focused largely on territory-holding adults during the breeding season. While the migration season has been studied to some extent (primarily north of the coterminous U.S. [7, 8]), little information is available on distribution and habitat selection during the winter season in interior western North America. In the core of this species’ range, winter distribution of resident adults with breeding territories is likely represented in available information on nesting habitat [9, 10], but the extent to which protecting nesting habitat also protects other eagle groups during winter is unknown. A conservation plan developed around breeding habitats may thus fail to adequately conserve birds that migrate to the same general area to overwinter, as well as resident non-breeding birds.

Threats to golden eagles also vary among seasons and life-history groups as a function of the dynamic spatial and temporal overlap in the distributions of both eagles and hazards [11, 12]. As such, some hazards affect eagles year-round (e.g., turbine strike mortality at wind energy developments), while others affect only one group of eagles during one season (e.g., disturbance of adult resident breeders and offspring at nest sites), and others have a disproportionately large impact during a specific season [e.g., lead poisoning from big-game hunting during fall [13]], collisions with vehicles while feeding on roadkill during winter [14, 15]. The population-level risk likely is greatest during winter when resident golden eagle populations in the lower-48 states are augmented by long-distance migrants from Alaska and Canada, resulting in exposure of a larger and more geographically diverse group of eagles to hazards during a period when they may be stressed from lower availability of live prey [15]. Risk to migrants and non-breeding residents could be further elevated to the extent that their winter distributions differ from the nesting habitat. Efforts to avoid risk to eagles (e.g., siting wind energy developments or retrofitting dangerous power poles) could thus be improved by accounting for areas of high-intensity winter use outside breeding habitat. Moreover, because regulations protecting golden eagles in the U.S. assign equal value to individuals regardless of geographic origin [16, 17], an improved understanding of winter distributions could help to reduce risk of liability for developers.

The state of Wyoming, USA, provides continentally important, year-round habitat for golden eagles [18, 19], but also includes a range of threats to the species. However, lack of information on the winter distribution of golden eagles is currently limiting full annual cycle conservation of both the year-round residents and long-distance northern migrants that winter in this region. To address this conservation problem, we developed models to predict spatial variation in intensity of use by wintering golden eagles in ecoregions that overlap with Wyoming, USA. Our specific objectives were to:

1. Develop models to predict spatial variation in relative intensity of use by golden eagles during winter for the life-history groups of the population in Wyoming not accounted for in existing models of nesting habitat.
2. Assess the reliability of predictions for decision making by conducting a thorough evaluation of model performance, including assessments by life-history groups of eagles, and geographic subregions.
3. Compare mapped predictions for the winter season with available nesting habitat models to quantify the extent and geographic pattern of overlap.

## Materials and methods

### Study area

**Fig 1:**
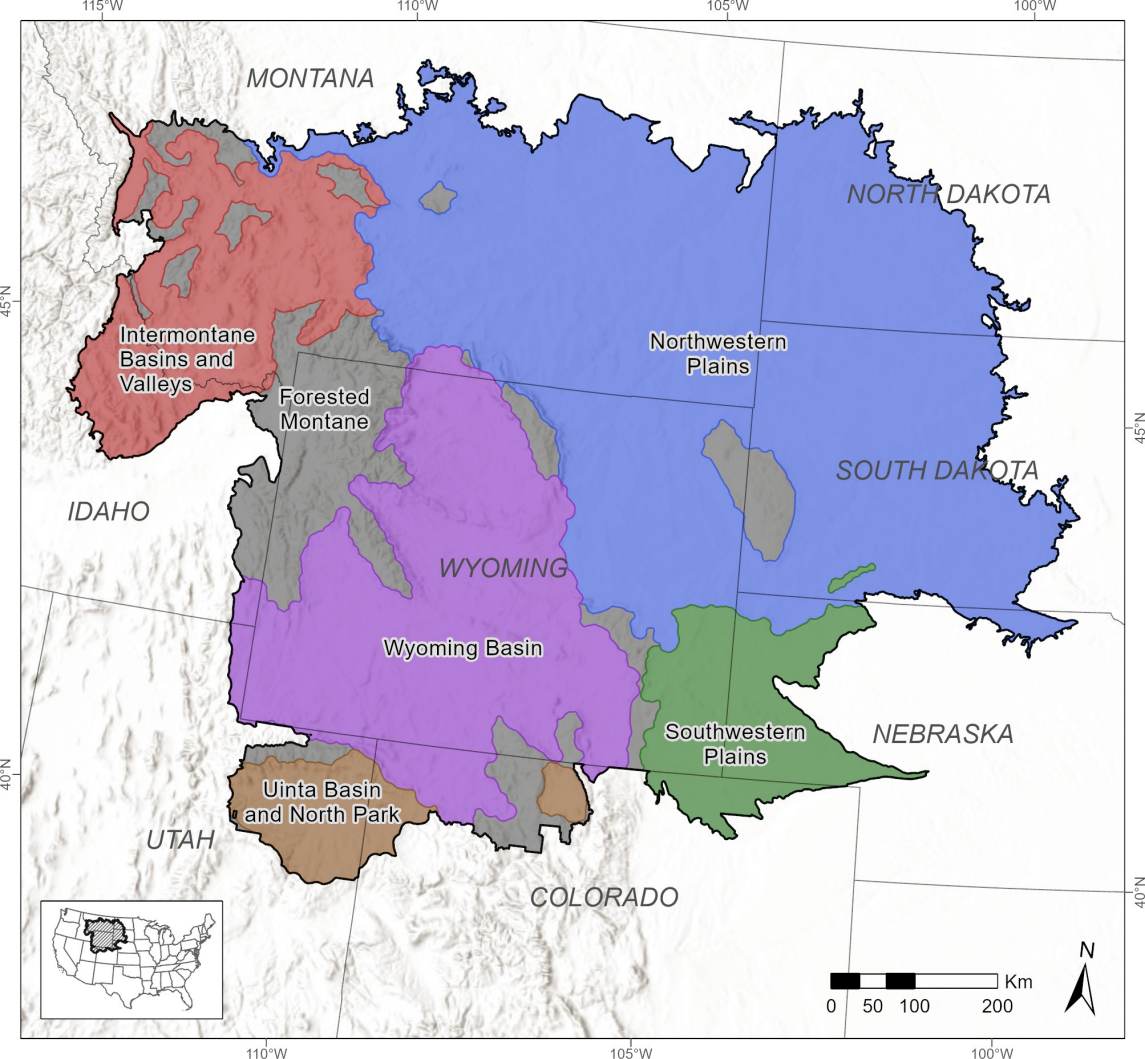
Study area for winter-season distribution modeling of golden eagles in Wyoming, USA, and surrounding ecoregions. Map shows study area (black outline), with boundaries of states (gray lines) and ecoregions (colored shading). Inset shows the location of the study area in the continental United States. Ecoregion data from the Commission for Environmental Cooperation [20] as modified by Dunk et al. [10], state boundaries from U.S. Census Bureau [21], and terrain base map modified from National Hydrography Dataset [22].

Our 765,953-km^2^ study area was based on level-III North American terrestrial ecoregions [20], as modified by Dunk et al. [10] to define modeling ecoregions relevant to golden eagles. Our study area consisted of those ecoregions that overlapped or were adjacent to state of Wyoming, USA, including the Forested Montane, Intermontane Basins and Valleys, Northwestern Plains, Southwestern Plains, Uinta Basin and North Park, and Wyoming Basin (Fig 1), to which we made some minor modifications (S2 File). We focused on Wyoming because it is among the most important areas for golden eagles in the western U.S., demonstrated by its high breeding-season density of golden eagles [19], extensive nesting habitat [10], and large numbers of long-distance migrants during winter [7, 18]. We included portions of the ecoregions outside Wyoming to improve predictions within the state by leveraging data from ecologically similar areas and to increase the extent of the area where our results could be used to inform conservation.

### Golden eagle location data

We acquired telemetry location data for golden eagles from across western North America that were instrumented with transmitters as part of at least 17 studies by 8 collaborator groups, including Federal, State, Tribal, and non-governmental organizations (S2 File). Transmitters included models that sent Global Positioning Systems (GPS) location data over mobile phone or ARGOS networks, and Doppler shift location data sent via the ARGOS network.

We used a series of filtering steps to select accurate locations that represented winter-season daytime sedentary locations of golden eagles from life-history groups not associated with breeding territories in our study area (Table 1).

**Table 1:** Outline of data filtering steps for telemetry locations used to model the winter-season distribution of golden eagles in Wyoming, USA, and surrounding ecoregions.

Step name	Filtering action	Goal
1. Initial data proofing	Remove erroneous and imprecise locations	Use accurate eagle locations for modeling
2. Season and time of day	Retain winter daytime locations	Focus model on daytime winter use
3. Life-history group	Remove locations associated with breeding-season home ranges	Focus model on life-history groups (Table 2) not represented in existing nesting habitat models
		Classify locations by life-history group for later evaluation
4. Behavioral state	Remove fast, long-distance movements and retain sedentary locations	Focus model on locations likely to be most representative of winter habitat selection, rather than migratory transiting movements
5. Temporal thinning	Retain 2 locations per day per individual	Reduce auto-correlation of telemetry locations
6. Test-train data selection	Split 75% for model training and 25% for testing	Train model on large dataset, while testing with observations not used in model development

Table shows the name of each step, the filtering action taken, and its goal. Detailed description and justification are included in paragraphs for each step name.

#### 1. Initial data proofing

To remove erroneous locations from the winter telemetry data, we visually inspected GPS fixes for obvious outliers, and retained Argos fixes in location classes 3, 2, and 1 (estimated error radii of <250 m, 250–500 m, and 500–1500 m, respectively [23, 24]) that filled in gaps in GPS data series. Fixes were filtered via the Douglas Argos-filter algorithm [25]. We removed fixes that resulted in spikes with angles < 15° and 25° lengths > 2500 m and 5000 m, respectively. We then passed fixes through velocity filters of 20, 27.8, and 40 m/s, generated tracks for each filtered set, and selected which velocity filter to use based on visual inspection of individual tracks. After filtering, we standardized the locations temporally by subsampling to a maximum of 1 location per individual per hour.

#### 2. Season and time of day

We attributed locations with season, time, and time of day. We used a conservative definition of the winter season as December-February to exclude most migratory movements in spring and fall. While the data used to test and train the model included only locations from the winter period, we used summer season locations to define breeding season home-ranges used as part of the data filtering process (see life-history groups section below), which we defined as June-August. We sought to develop a model that would accurately predict daytime use, so we removed locations at night and within one hour of sunrise or sunset based on the location and timestamp. To do this, we used python to implement the National Ocean and Atmospheric Administration Solar Calculation method [26], which is based on astronomical algorithms.

#### 3. Life-history groups

Our objective was to complement existing models of nesting habitat [10] with a model of winter-season use by eagles that was not associated with local breeding territories. Thus we included migrants, non-migrant non-territorial eagles of all ages, and locations outside nesting territories from adult non-migrant territory holders (i.e., resident breeders) and their young. To accomplish this, we used a multi-step process to classify individuals by age, migratory status, and territory status in our study area (Table 2), and annotated their locations with those attributes. We then removed locations associated with known or suspected breeding territories within our study area. Age classes were assigned using a biological year starting in April, when eggs typically hatch in our study area. We used the following steps to classify and select data for analysis:

**Table 2:** Sample sizes of golden eagle telemetry locations used to model winter-season distribution of golden eagles in Wyoming, USA, and surrounding ecoregions.

Name	Age	Migratory Status	Territory Status in Study Area	N locations (N deployments)		
				Daytime		Night roost
				Training	Test	Test
Adult migrants	Adult	Migrant	Non-territorial	5290 (54)	1785 (51)	3575 (53)
Adult resident “breeders” ^a^,^b^	Adult	Non-migrant	Territory holder	4889 (60)	1620 (57)	1092 (58)
Adult resident “floaters” ^b^	Adult	Non-migrant	Non-territorial	3052 (44)	972 (43)	7173 (72)
Non-adult residents ^a^	Non-adult	Non-migrant	Non-territorial	9678 (74)	3220 (70)	6284 (88)
Non-adult migrants	Non-adult	Migrant	Non-territorial	3581 (36)	1232 (34)	2021 (31)

Table shows the number of daytime training and test locations, and night roost locations by age, migratory status, and territory status, with number of individual deployments is shown in parentheses.

^a^ Excluding locations of resident adult territory holders within breeding territory polygons and ≤1 year-old non-adults within 3.2 km of natal site (see life-history group classification methods for details).

^b^ Adult resident “breeders” and “floaters” were combined for model evaluations.

First, we defined migrants as individuals that originated or had a summer home range north of 58.25͒ N latitude, and retained all winter locations within our study area, regardless of age. We visually inspected all tracks to confirm that no birds tagged as nestlings in the study area migrated north during the following year. All winter locations from these individuals were relevant for our analysis because they could not have had breeding home ranges in our study area. Individuals ≥4 years old were classified as **Adult Migrants** and <4 years as **Non-adult Migrants**.

For eagles tagged as nestlings or fledglings with known natal sites in the study area, we removed locations that were both within 1 year of tagging and ≤3.2 km of the natal site (“territory core area” radius from [27]) because these locations were associated with known breeding territories. For eagles tagged in their first calendar year of life with unknown natal sites (n = 10), we estimated annual 95% kernel density estimator (KDE; Worton method [28] with proportion of 0.7) home range polygons for the summer and winter seasons and removed all locations for individuals that had overlapping, small (<200 km^2^) seasonal home ranges (n = 2) because we could not be certain the overlap was not related to their natal territory. This was a conservative approach to make sure we removed locations of 1-year old eagles that could have been in their natal territory. Individuals in this group were classified as **Non-Adult Non-migrants**.

For 2-3-year-old eagles not tagged as nestlings, we retained all winter locations within our study area because they were too young to be associated with their own breeding territory and the likelihood they returned to a natal territory was minimal [24]. Individuals in this group were also classified as **Non-Adult Non-migrants**.

For adult and near-adult eagles (≥4 years old), we retained all winter locations for individuals that had all summer locations >16 km outside the study area because that indicated they did not have a breeding territory in the study area. If any summer locations for an individual were within 16 km of the study area, we estimated annual 95% KDE home range polygons for the summer season, removed locations within the home range, and classified the remaining locations as **Adult Non-migrant Non-territorial**. We removed all points for individuals in this group with inadequate data to assess potential summer territories (n = 5), which we defined as those with KDEs created from <25 locations and/or <30 days.

The remaining adult individuals had summer home ranges that were within or substantially overlapped our study area. We considered any bird in this group with a home range size <200 km^2^ to be a territory holder. While our dataset and other studies (e.g., [29]) suggested home ranges of known breeders within our study area were <100 km^2^, we chose a more conservative threshold of 200 km^2^ to ensure that we did not include locations from within breeding home ranges. We retained all winter points for adult eagles with summer home ranges >200 km^2^, indicating non-territorial status, and classified them as **Adult Non-migrant Non-territorial** (i.e., adult resident “floaters”). For adult eagles with summer home ranges <200 km^2^, we excluded winter locations that fell within individuals’ previous summer KDE, or the following summer KDE if the previous summer was not available. We retained locations from outside the KDE home ranges and classified them as **Adult Non-migrant Territory-holders** (i.e., adult resident “breeders”).

#### 4. Behavioral state

We used the residence in space and time (RST) method to classify points as associated with either “sedentary” or “transiting” movements. The RST algorithm uses the time spent in a circular area around each point to classify them as distance-intensive (i.e., transiting), or time-intensive and time- and distance-intensive (i.e., sedentary [30]). To ensure adequate assessment of movement modeling, we considered only tracking bouts of ≥28 days with no gaps >48 hours and modeled discontinuous tracking segments from the same individual separately. We visually evaluated a range of radii (6, 10.5, 15, 22.5, and 30 km) and found the results to be similar. Accordingly, we selected the largest radius (30-km) for an inclusive definition of sedentary behavior that resulted in removal of only the fastest, long-distance transiting movements. We selected for subsequent analysis only sedentary points because we expected they would have the strongest signal of habitat use for eagles localized in wintering ranges, while transiting points would represent longer distance directed movements (e.g., between foraging areas) in conditions resembling migratory habitat.

#### 5. Temporal thinning

To reduce spatial and temporal autocorrelation of data points, we randomly sampled 2 locations per individual per day. We included 1 in the morning and 1 in the afternoon that were separated by a minimum of 1 hr based on dividing the day by solar noon for the study area during winter (ca. 1220 hrs).

#### 6. Final test-train data selection

From this final filtered dataset, we randomly selected 75% of locations to train the model and withheld 25% of locations for model testing/evaluation. As part of our model evaluation, we used the life-history classifications of the filtered dataset to assess whether all subgroups of golden eagles showed the same general patterns of spatial variation in intensity of use. Additionally, we used a separate dataset of nocturnal roost locations to evaluate the ability of the model trained on daytime sedentary locations to predict variation in the relative intensity of use of nocturnal roosting locations. This dataset consisted of one roost location after civil twilight and closest to 2400 hrs for each bird and night available.

### Model development

The primary goal of our analysis was to make accurate predictions to support conservation planning, and secondarily to understand ecological relationships of golden eagles [31]. Accordingly, we developed models using a flexible, multi-stage process that emphasized tuning and evaluation. We selected from a large set of candidate predictors, fitted models with a machine learning algorithm (MaxEnt [32]), used a tuning process to minimize both over- and under-fitting, then conducted an extensive set of evaluations to quantify the predictive performance of the model for different golden eagle life-history groups and distinct geographic regions of our study area. To capture all relevant life-history groups with the minimum number of models, we first developed a combined model using the pooled data for all life-history groups, which we then evaluated to determine if separate models were warranted for any groups.

#### Response variable

The MaxEnt algorithm is a machine learning model that relates presence and background (i.e., used and available) locations to geospatial variables to generate a continuous predictive surface (i.e., heat map) of species distribution [32]. MaxEnt is a count-based model that is equivalent to an inhomogeneous Poisson point process [33]. The response variable of MaxEnt models trained on locations of unique individuals can be interpreted as the relative probability of occurrence for the species [33] and technically represents that relative density of locations [10]. Because we trained our model on thinned GPS telemetry data that included multiple use locations per individual, we interpreted the response variable as the relative intensity of use by the species. This terminology is consistent with other count-based models of resource selection for animal movement data (e.g., [34]). MaxEnt, resource selection functions, and other models of species distribution are also commonly referred to as Habitat Suitability Models because they relate occurrence data to geospatial variables that represent characteristics of habitat [35]. Accordingly, we refer to the response variable of our model as “relative intensity of use” and also describe the modeled predictions in terms of the distribution, amount, and relative value of winter “habitat”.

#### Predictor variables

We compiled a library of environmental variables we predicted would affect golden eagle habitat selection during winter, consisting of 67 base variables from the categories of climate and weather (e.g., precipitation, snow depth, temperature; n = 71 variables total), vegetation and landcover (e.g., proportional cover of vegetation types, greenness indices; n = 366 variables total), developed areas (e.g., proportional cover of roads and urban areas; n = 24 variables total), topography (e.g., aspect, landforms, topographic indices; n = 185 variables total), wind and uplift (e.g., height of planetary boundary layer, orographic uplift, wind speed; n = 86 variables total), and ecoregions (6 ecoregions as categorical variable; n = 1 variable total) (S1 File). Geospatial data for all variables were resampled to 120-m resolution raster grids, then summarized at ≤6 spatial extents (120 m to 6.4 km) using a moving window approach and ≤4 focal statistics (mean, SD, min, max) appropriate to each variable. The range of spatial extents was chosen to capture the scales of habitat selection by golden eagles, from the habitat conditions in the immediate vicinity of a nest site or use location to the broader extent of a nesting territories or migratory movements. This resulted in the estimation of a relatively large number of variables (>700) that were necessary to optimize the spatial extent and focal statistic for each environmental predictor in the limited set of base variables. We suggest that our set of base variables (n = 67) was of a reasonable size to describe the potential ecological relationships of golden eagle habitat use over a large study area, and we interpret the selection of scale and focal statistic for each variable as an optimization step rather than variable selection per se. Although all animal locations were within the study area, for extraction of covariate values and model projections, we added a 20-km buffer around the study area to reduce bias near the edges of the area from missing data in moving window summaries.

We used a three-step process to reduce the number of predictor variables included in the MaxEnt model based on the methods of Dunk et al. [10]. 1) We selected the “best” neighborhood extent and focal statistic among the covariates derived from each base variable. To do this, we computed the ratio of the mean value at training locations to the mean value at a random sample of locations (n = 10,000) and retained the covariate with the largest ratio and <20% of locations with non-zero values. Dunk et al. [10] found that screening predictors using 10,000 locations was an effective and computationally efficient approach to reduce the candidate set of variables before fitting models. For competitive variables, we used our judgment to select the scale and focal statistic most consistent with golden eagle ecology, generally selecting those with medium neighborhood extents and more easily interpretable focal statistics (e.g., mean over SD). 2) We removed redundant variables from the set of predictors selected in step 1. To do this, we computed variance inflation factors (VIF) among the predictors separately by variable category and removed variables with VIF ≥4. 3) We fitted an initial model (described below) with all the covariates retained from step 2, then removed variables with <1% contribution in multiple runs (2 or 3) until no variables had <1% contribution.

Ecological inference is a common secondary goal of species distribution analyses aimed primarily at spatial prediction, and models with predictors grounded in species’ ecology are expected to produce fewer spurious relationships and have better transferability [36, 37]. Some mechanistic interpretation of relationships in distribution models is thus justified, with the caveat that the correlations do not imply causation. Given the wide range of functional forms and interactions explored by the MaxEnt algorithm, we further limited our ecological interpretation of variables to their relative importance, and the spatial scale and general direction of their relationships to golden eagle use.

#### Model fitting, tuning, and projection

We fitted models using the MaxEnt model algorithm [32]. For all covariates, we evaluated interactions and multiple functional forms, including linear, quadratic, product, threshold, and hinge. To reduce sampling bias from areas where telemetry data were not collected [32, 38], we used 100,000 background (i.e., “available”) points from an area within 20 km [10] around the training locations (hereafter the “modeled area”; S1 Fig). To avoid over- or under-fitting the model, we optimized the regularization multiplier in MaxEnt following the methods of Dunk et al. [10]. The default regularization multiplier in Maxent is 1.0, and predicted distribution maps are more localized for smaller values and more diffuse for larger values. The optimization process seeks to balance the trade-offs between tightly-fitted models that provide more accurate local predictions in areas with training data and more general models that have better transferability to areas without training data [37, 38]. After developing and evaluating the model within the modeled area, we projected it to the buffered study area, then clipped the predicted surface to the study area boundary [38]. Our projection step was similar to what Elith and Leathwick [36] term “model-based interpolation to unsampled sites”, in which predictions are made for the same time period and range of ecological conditions as the sampling area, in contrast to projecting a model to a new spatial or temporal frame (i.e., out of sample prediction).

### Model evaluation

We used three methods to assess the overall performance of the model. 1) Using the 25% of locations withheld from the training data, we compared number of use locations predicted by the model to those observed in the test data. To do this, we used the model to predict the number of locations in each of 10 equal-interval bins of relative intensity of use following the methods of Dunk et al. [10], then calculated the coefficient of determination (R^2^) between the observed and predicted number of locations in each bin. We interpreted higher values to indicate better fit. 2) Using the 25% of locations withheld from the training data, we evaluated the extent to which the distribution of test locations differed from random expectation under the model’s predictions using the Boyce Index [39, 40]. To do this, we estimated the area adjusted frequencies (AAF) of the evaluation data locations in each of 10 equal-interval bins of relative intensity of use, then calculated the Boyce Index as the rank correlation between the AAF of the bins and the bin ranks. We interpreted values of the Boyce Index >0.90 to indicate good performance of the model for a group. 3) Using the training data, we conducted a 10x cross-validation. This evaluation was similar to the first method described above, except that it used random sub-samples of the training data instead of the withheld data. For each of 10 iterations, we randomly selected approximately 25% of the training data, refitted the model, then compared the observed and predicted number of locations in 10 equal-interval bins of relative intensity of use. Although this step was done originally as part of our method to optimize the regularization multiplier (described above), we report the results along with our overall model evaluation as further evidence of model performance. We chose to use 10 equal-interval bins of relative intensity of use for most evaluations as a compromise between having too few bins to gain insights into the relationship between AAF and bin ranks and the magnitudes of differences among AAF values of bins, and having too many bins, which would have resulted in higher bins containing few to no locations and representing exceedingly small portions of the study area.

#### Geographic evaluation

To provide end users with a transparent assessment of the modeled predictions across our large study area, we evaluated geographic variation in model performance [41] within two landscape classifications: golden eagle modeling ecoregions (n = 6 regions, ~28,000–365,000 km^2^ [10]) and subregions based on USFS Ecological Sections (n = 15 subregions, ~10,000–94,000 km^2^ [42]). We used the evaluation approach described above (method 1) to compare the predicted number of use locations in each of 10 equal-interval bins within geographic regions to those observed in the withheld data.

To provide a finer-scale depiction of spatial variation in model performance, we compared the concordance among quartile bins of observed and predicted values within the cells of 30x30-km and 15x15-km grids overlaid on the modeling area. For each grid cell, we estimated the number of winter test locations in the cell by multiplying the mean AAF of the cell by the proportion of the modeling region the cell represented and the total number of winter test locations. We then divided those predicted numbers into quartile bins and compared them to quartile bins of the observed number of locations in each cell. We interpreted cells with matching bin ranks as accurately predicted and differences of 1–3 quartiles as representing low, medium, and high levels of discordance, respectively.

#### Life-history group evaluation

To assess the performance of our model for the different life-history groups in the dataset, we used the methods described above to compare the intensity predicted by the model to that observed in the test data (method 1) and calculated the Boyce Index (method 2) for each life-history group. We interpreted higher values of R^2^ between the observed and predicted number of locations to indicate better fit of the combined model across life-history groups and values of the Boyce Index >0.90 to indicate good performance of the model for a group. Additionally, we estimated the magnitude of the difference between the values of the highest and lowest AAF bins as an indicator of maximum difference in relative intensity among bins, and used the AAF ratio to assess whether the magnitude of difference was similar among life-history groups (method 3). We interpreted a magnitude of difference in highest:lowest bin AAF of >25 to indicate good performance of the model for a group based on ratios of previously published distribution models for golden eagles [10]) and other species (Northern Spotted Owl, *Strix occidentalis caurina* [43]; red-tree vole, *Arborimus longicaudus* [44]; fisher, *Martes pennanti* [45]). It was possible that we would find a model with a large Boyce Index and a small magnitude of difference in highest:lowest bin AAF. If so, we would consider creating a new model for that sub-group of eagles and not assume that the combined winter model adequately represented their spatial distribution during winter. The magnitude of the difference in AAF is also an indicator of the relative strength of selection, which we interpreted to indicate differences in habitat selection among life-history groups.

#### Independent data evaluation

We used an independent dataset of golden eagle GPS telemetry locations from within our study area (S1 Table) to test the ability of the model to predict the distribution of locations from new individuals. We used these data as an independent test dataset because they were not available until after our model had been developed and covered only portions of our study area. To do the evaluation, we processed the independent data as described above for the model training data, then compared the intensity predicted by the model to that observed in the independent data (method 1) and its difference from random expectation using the Boyce Index (method 2).

#### Nocturnal roost evaluation

As a final step, we tested the transferability of our model trained on daytime use locations to predicting the relative intensity of use of nocturnal roost locations aggregated across all individuals within each life-history group. To do this, we used the same methods as the other evaluations to compare the intensity predicted by the model to that observed in the test data (method 1) and calculated the Boyce Index (method 2) for each life-history group. We included this evaluation because we thought it would be valuable for land managers to know whether our model trained and tested on daytime use locations also accounted for spatial variation in nocturnal roost locations or if a separate model would be needed for spatial prioritization of that habitat component.

### Summary and comparisons

#### Overlap with nesting habitat

To quantify differences in the distribution of wintering and nesting habitat, we overlaid the winter model predictions with the ecoregional golden eagle nesting habitat models of Dunk et al. [10]. The nesting habitat models used a similar overall modeling approach to our winter-season model, except the training data were nest locations and separate models were developed for each ecoregion [10]. We calculated the Spearman correlation between the overlapping area of the predictive surfaces for each ecoregional nesting habitat model and the winter model as a measure of their overall similarity, and the percent overlap of the area within the top 10% and 20% quantiles as a measure of the degree to which the models agreed in areas of high predicted use.

#### Surface management

To describe the model predictions and help identify where opportunities exist for management and conservation, we summarized the percentage of high-use eagle habitat (top 10% and 20% quantile areas of predicted intensity of use) within administrative categories (i.e., surface management entities). As an index of the concentration of high-use habitat among land management categories, we calculated the ratio of observed to expected habitat by dividing the percentage of the top 10% bin by the percentage of the study area composed by each category. Values of the observed:expected ratio >1 indicated more high-use habitat than expected given the size of an administrative area, while values <1 indicated less.

## Results

The raw golden eagle telemetry dataset compiled for our study area consisted of 659,521 locations from 344 deployments spanning 2006–2020. The filtered dataset comprised 35,319 locations from 203 individuals, with each individual contributing an average of 2.69 years of data (SD = 1.76) and 131 locations (SD = 126, range = 1–784). From the filtered dataset, we retained 26,490 locations (75%) for model training and 8,829 locations (25%) for testing (Table 2). The final dataset was comprised of 91% GPS telemetry and 9% Doppler shift locations.

We began model development with 67 base variables, which estimated for multiple spatial extents, summary statistics, and seasons, resulted in 733 total candidate variables. Following steps 1–2 of our variable reduction process, we retained 63 covariates for inclusion in the initial model. After dropping covariates that contributed <1% (step 3), the final model included 17 covariates (Table 3 and S2 Fig). The covariates contributing to the final model were wind and uplift (40.2% contribution), vegetation and landcover (27.7%), topography (14%), climate and weather (9.4%), and ecoregion (8.7%). Variables at the finest scale (120-m focal extent) contributed the most (42.5%), with approximately half of contributions from variables summarized at extents of ≤1 km (56.8%) or ≥2 km (43.2%).

**Table 3:** Covariates included in the final model of winter-season distribution of golden eagles in Wyoming, USA, and surrounding ecoregions.

Variable Category	Base variable Name	Focal Extent	Focal Statistic	Contribution (%)
Wind and uplift	Orographic uplift index (Winter)	120 m	Mean	22.0
Wind and uplift	Height of planetary boundary layer (Winter)	32 km	Mean	14.6
Ecoregion	Ecoregion	30,100–388,988 km^2^	NA	8.7
Vegetation and landcover	Normalized difference vegetation index (Spring)	120 m	Mean	6.6
Topography	Terrain Ruggedness Index	120 m	SD	6.4
Climate and weather	Snow depth (Winter)	500 m	SD	5.1
Vegetation and landcover	Proportion of cool semi-desert scrub and grassland landcover	1 km	SD	4.8
Vegetation and landcover	Proportion of shrub and scrub landcover	500 m	Mean	4.4
Topography	Proportion of steeply sloping landforms	6.4 km	Mean	4.4
Climate and weather	Mean annual precipitation amount	120 m	Mean	4.3
Vegetation and landcover	Proportion of tall sagebrush landcover	2 km	SD	4.2
Wind and uplift	Thermal uplift index (Winter)	3 km	Mean	3.6
Vegetation and landcover	Proportion of cottonwood tree landcover	2 km	Mean	2.8
Vegetation and landcover	Percent herbaceous canopy cover	3.2 km	SD	2.6
Vegetation and landcover	Proportion of alfalfa cropland landcover	3.2 km	Mean	2.3
Topography	Local elevational difference	120 m	SD	1.9
Topography	Topographic wetness index	120 m	SD	1.3

**Fig 2:**
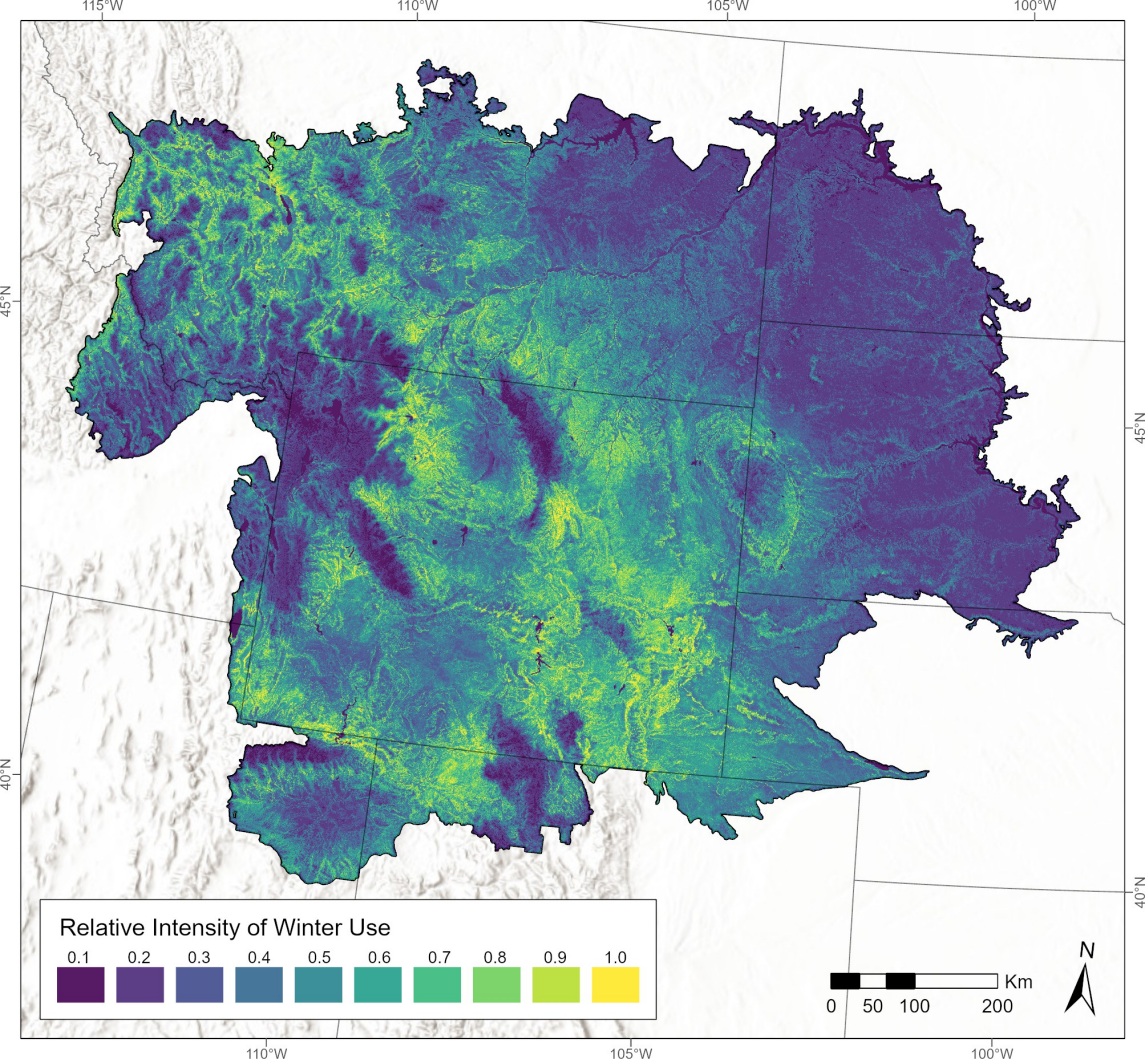
Map of spatial variation in the relative intensity of daytime winter use by golden eagles in Wyoming, USA, and surrounding ecoregions. State borders and topographic shading are shown in gray. Color scale for intensity of use is labeled with the upper bounds of 10 equal-interval bins from 0.0–1.0. State boundaries from U.S. Census Bureau [21] and terrain base map modified from National Hydrography Dataset [22].

The optimized regularization multiplier of 4.0 was greater than the default of 1.0, suggesting a relatively more general output distribution achieved the best balance between over- and under-fitting. When projected to the entire study area, the final model predicted large areas with relatively low-intensity winter use by golden eagles and a smaller area of relatively high-intensity use that was concentrated in Wyoming (Fig 2). Areas with higher predicted intensity of use occurred in portions of the Wyoming Basin, including the upper Powder, upper Wind River, western Bighorn, Shirley, and Laramie Basins, and the extent of Wyoming Basin in Colorado, southwestern Wyoming, the southern Great Plains of southeastern Wyoming, and the intermontane valleys of western and central Montana.

### Model performance

**Fig 3:**
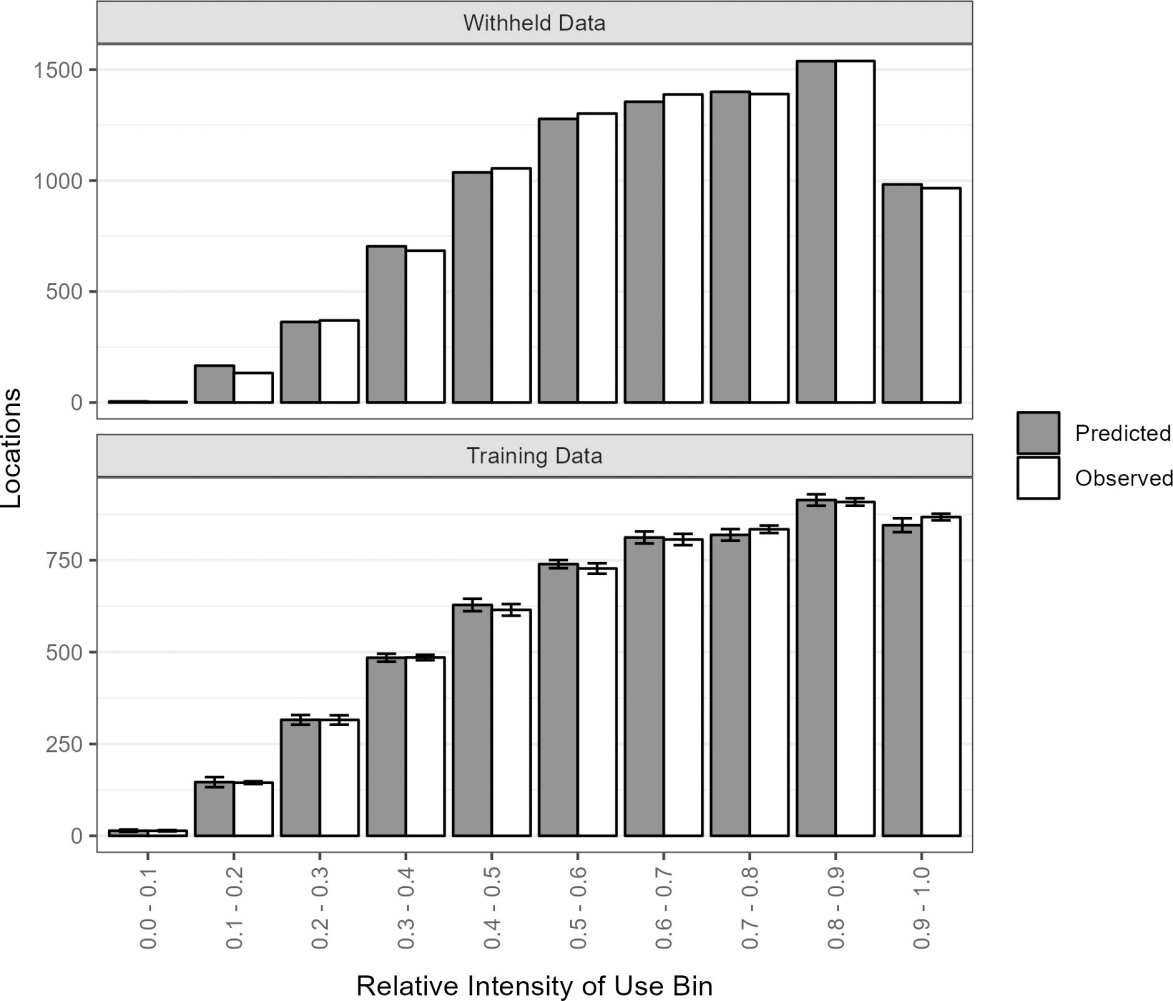
Bar graphs of predicted and observed numbers of winter locations of golden eagles in each of 10 equal-interval bins of relative intensity of use for 25% of locations (n = 8,829) withheld for model testing (upper panel) and cross-validation using 22% (n = 5,719) of training data points randomly selected 10x (lower panel).

The final model made accurate predictions of the withheld test data, with average deviation between observed and predicted values across all bins of 7.3% (SD = 12.9%), and ≤2.8% for the top 8 bins (Fig 3). The rank-correlation of bins (Boyce Index) was 1.00, indicating the model classified the withheld data well. The 10x cross-validation using the training data also suggested a high degree of accuracy, with narrow and overlapping 95% CI for all bins (Fig 3).

#### Geographic subregions\

**Fig 4:**
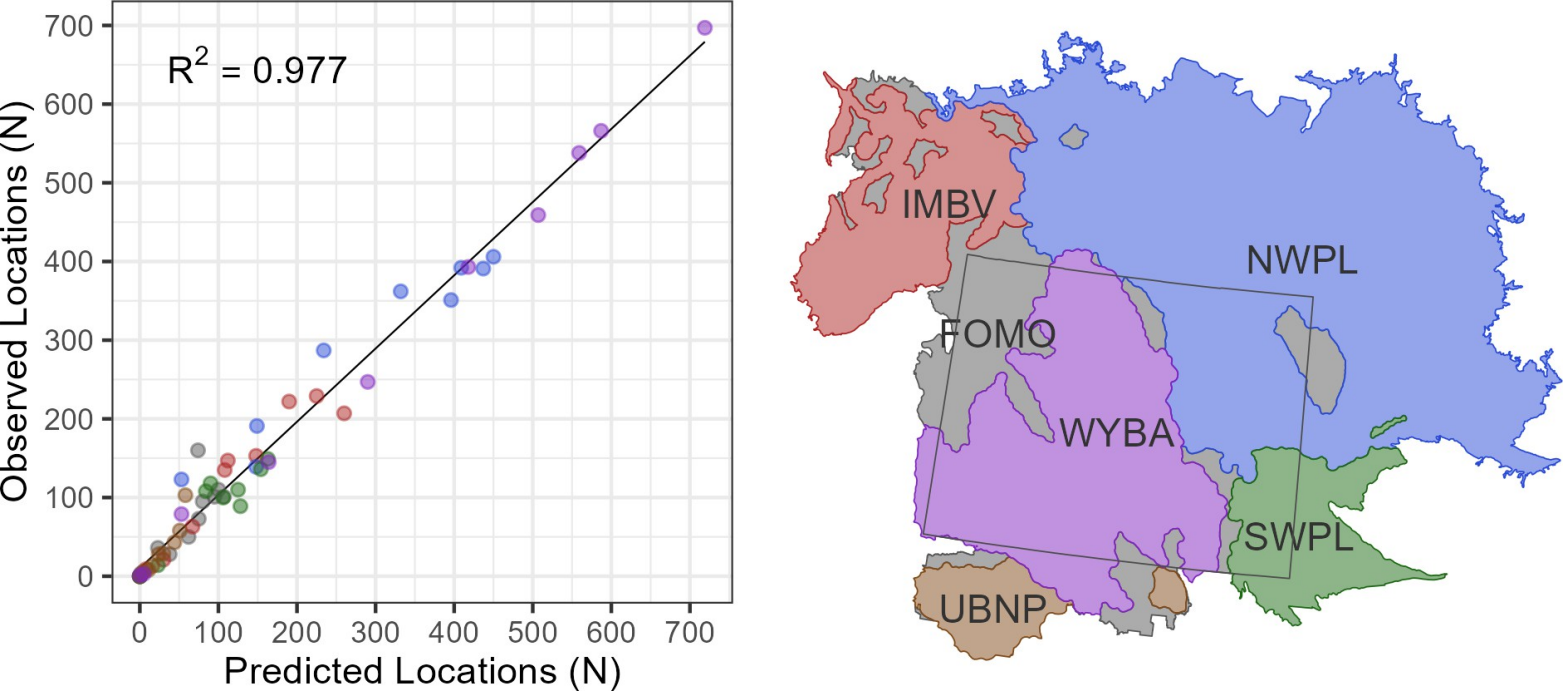
Scatterplots of predicted versus observed numbers of golden eagle winter locations among 10 equal-interval bins of relative intensity of use within 6 ecoregions. Ecoregion codes are Forested Montane (FOMO), Intermontane Basins and Valleys (IMBV), Northwestern Plains (NWPL), Southwestern Plains (SWPL), Uinta Basin and North Park (UBNP), and Wyoming Basin (WYBA). Ecoregion data from the Commission for Environmental Cooperation [20] as modified by Dunk et al. [10] and state boundaries from U.S. Census Bureau [21].

The model accurately predicted relative intensity of use within geographic subregions for both the larger ecoregions (R^2^ = 0.977; Fig 4) and smaller ecological subregions (R^2^ = 0.91; S3 Fig). The grid-based evaluations showed that the model’s predictive accuracy decreased with spatial scale, with greater concordance among quartile bins for the 30x30-km grid evaluation than the 15x15-km grid. For both grids, the highest degree of concordance was found for the lowest (90.28% for the 30x30-km grid) and highest (59.63% for the 30x30-km grid) quartiles, while most discordant predictions were off by only one quartile (S4 Fig). Among the four quartiles in the 30x30-km grid, the proportion of cells that were discordant by two quartiles ranged from 0.028 to 0.224, while the proportion discordant by three quartiles ranged from 0.014 to 0.101 (S4 Fig).

#### Independent test data

**Fig 5:**
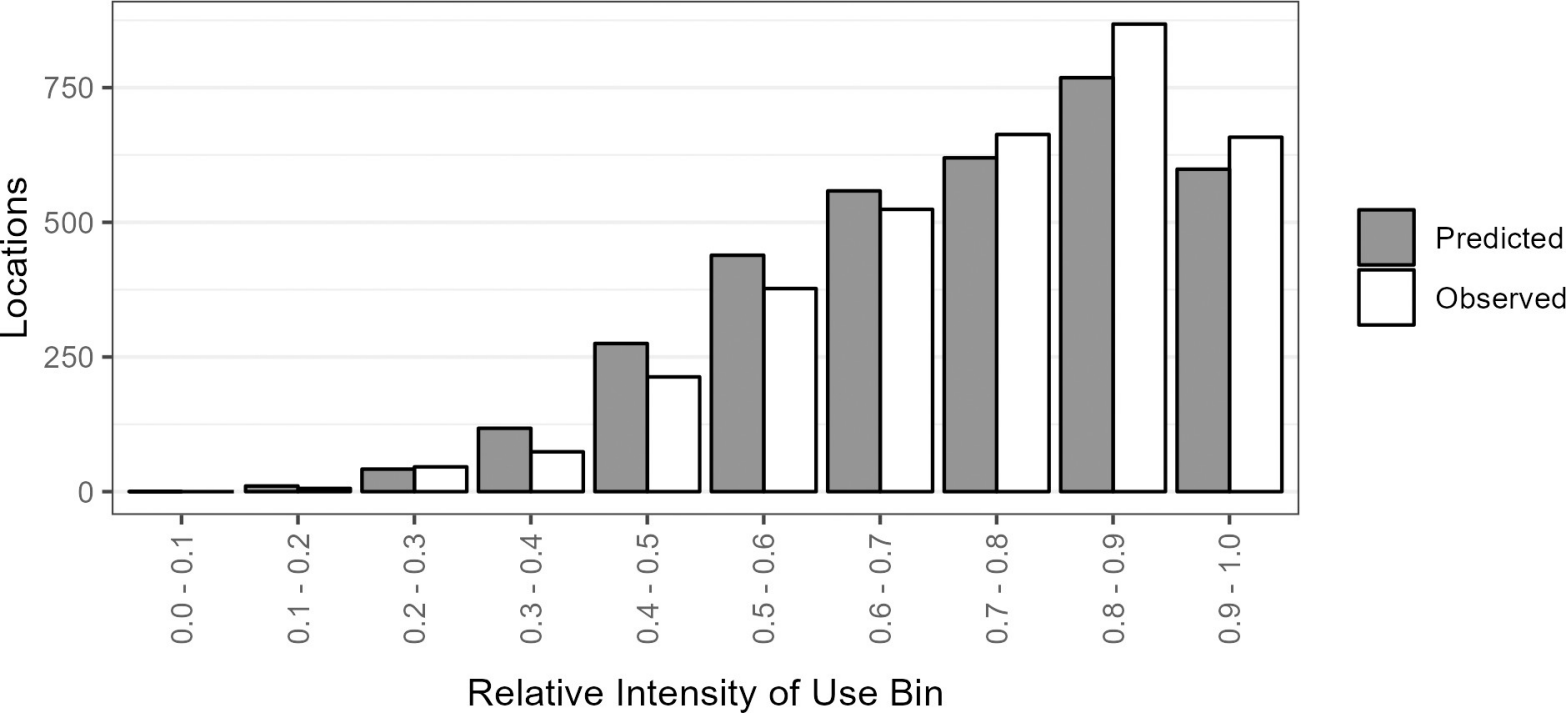
Predicted and observed numbers of independent golden eagle locations in each of 10 equal-interval bins of relative intensity of use.

The independent test dataset included 191,219 locations from 46 individuals, which our filtering process reduced to 3,429 locations from 24 individuals spanning 2017–2020. The test area was 118,435 km^2^ (15% of the study area), centered in south-eastern Wyoming and including an area of western Montana. Our model accurately predicted the distribution of the independent test data among bins, with average deviation between observed and predicted values across all bins of 25% (SD = 25%), and ≤11% for the top 3 bins (Fig 5). The rank-correlation of bins (Boyce Index) was 1.00, indicating the model classified the independent data well.

#### Life-history and behavioral groups

**Fig 6:**
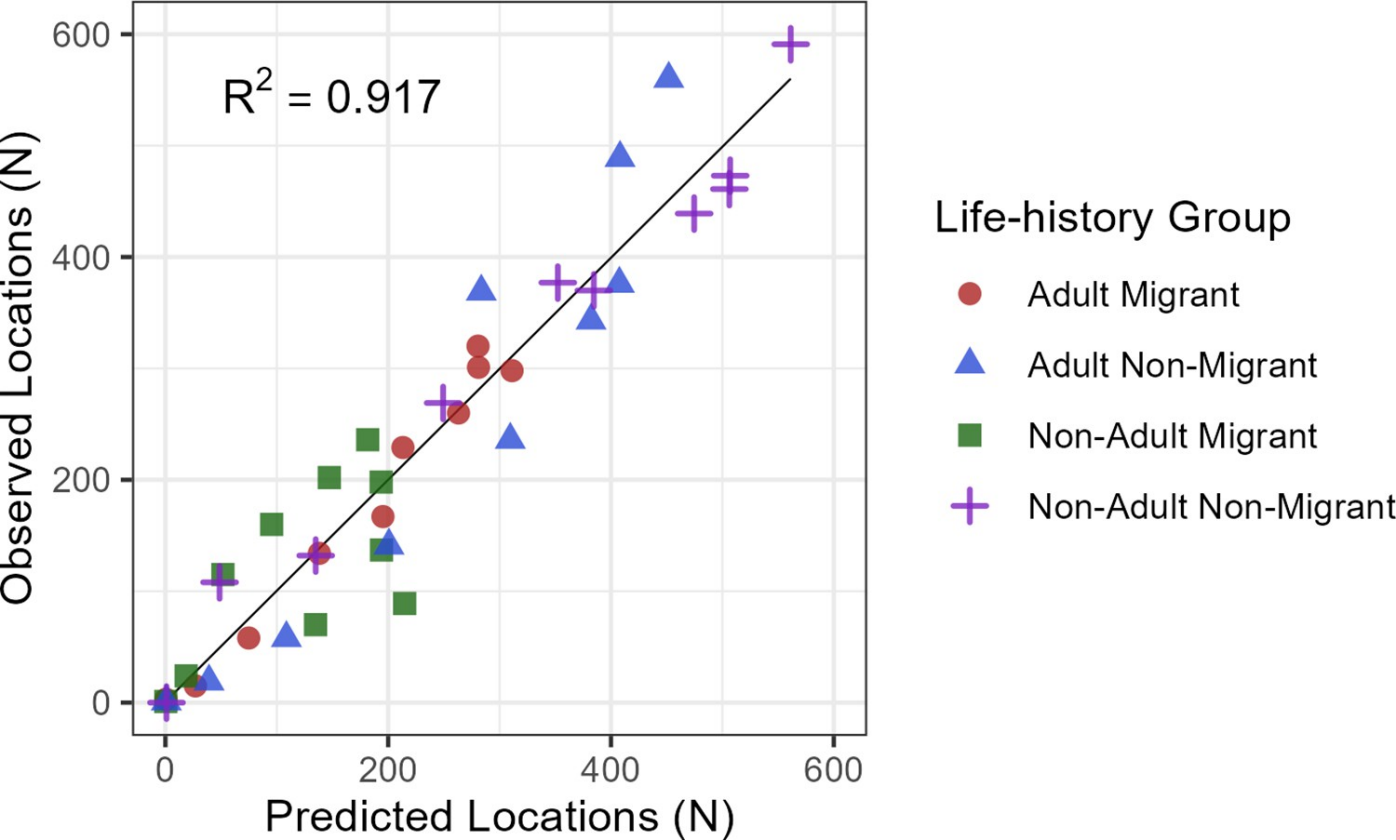
Scatterplot of observed versus predicted numbers of winter locations of golden eagles by life-history group among each of 10 equal-interval bins of relative intensity of use for the 25% of locations (n = 8,829) withheld from the model training.

The model accurately predicted relative intensity of use for life-history groups, based on the correlation of observed and predicted values (R^2^ = 0.917; Fig 6) and the rank-correlation of area adjusted frequencies (≥0.97; Table 4). Thus, we interpreted the single model as adequate for all life-history groups and did not create separate models for any groups. The magnitude of the difference in intensity between the highest and lowest AAF bins was greatest for adult non-migrants (854), suggesting the best model performance for that group, followed by non-adult (162) and adult (129) migrants, and smallest for non-adult non-migrants (51). The model, which was developed with daytime sedentary locations, was also a relatively good predictor of nocturnal roost locations across life-history groups (R^2^: 0.793), with the greatest deviation for the adult non-migrant group (S5 Fig).

**Table 4:** Area-adjusted frequencies (AAF) and ranks of winter locations of golden eagles by life-history group among each of 10 equal-interval bins of relative intensity of use for the 25% of locations (n = 8,829) withheld from the model training.

Bin	Adult Migrant		Adult Non-Migrant		Non-Adult Migrant		Non-Adult Non-Migrant	
	AAF	Rank	AAF	Rank	AAF	Rank	AAF	Rank
1	0.077	2	0.018	1	0.037	1	0.000	1
2	0.060	1	0.053	2	0.140	2	0.241	3
3	0.186	3	0.128	3	0.536	3	0.235	2
4	0.412	4	0.298	4	0.712	4	0.458	4
5	0.794	5	0.564	5	1.015	5	0.711	5
6	1.154	6	1.048	6	1.518	6	1.080	6
7	1.980	7	1.602	7	1.775	7	1.581	7
8	2.765	8	3.093	8	1.823	8	2.409	8
9	4.989	9	6.456	9	2.159	9	5.485	9
10	9.890	10	15.049	10	6.006	10	12.376	10
R^2^ (Boyce Index)		0.976		1.000		1.000		0.976
AAF magnitude of difference	129		854		162		51*	

The correlation of the bin ranks (Boyce Index) indicates the extent to which the distribution of test locations differed from random expectation under the model’s predictions. The AAF magnitude of difference indicates the discriminative ability of the model for each group.

* Ratio of bins 10:2 because no locations were in lowest bin.

### Overlap with nesting habitat

**Fig 7:**
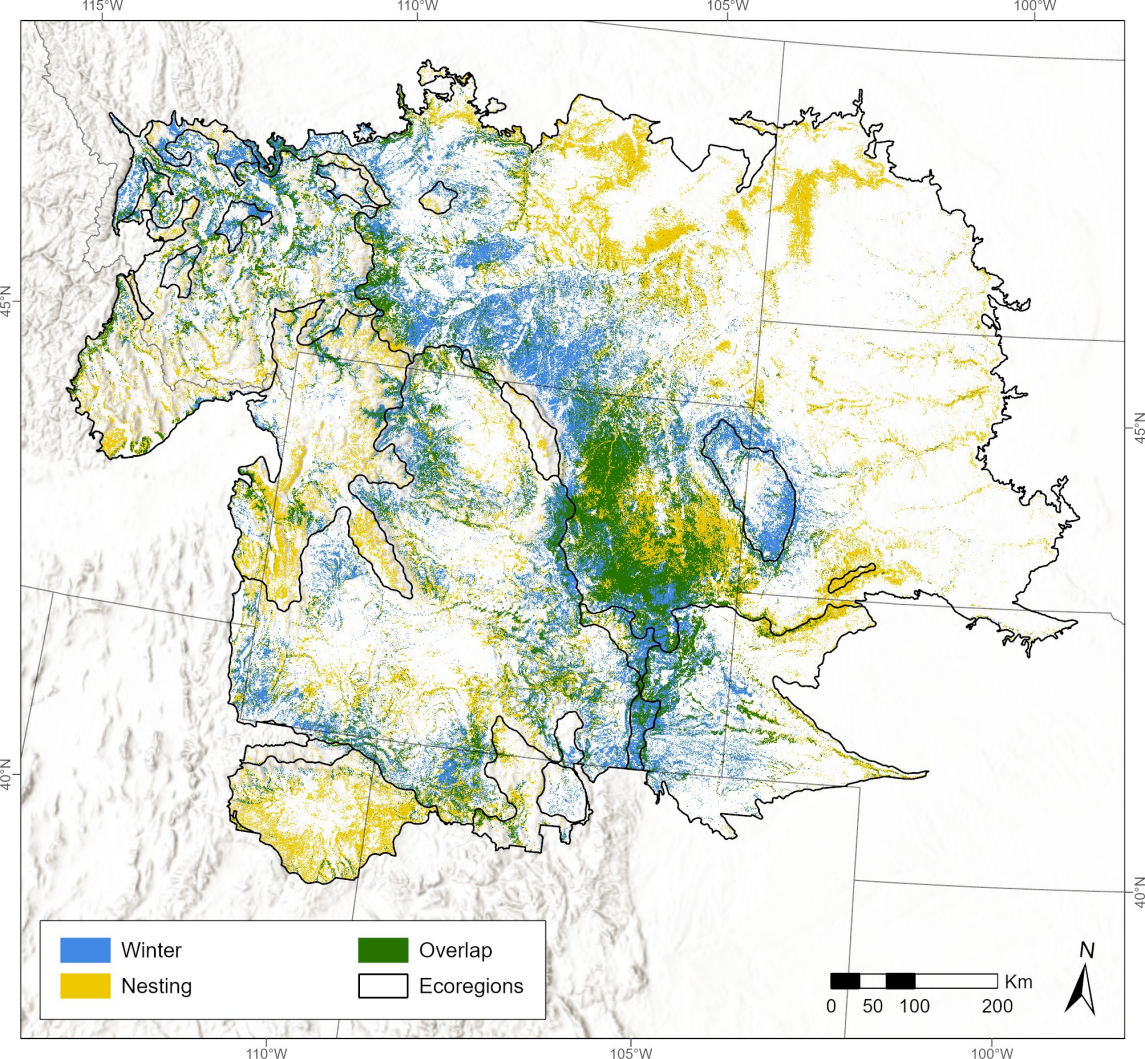
Overlap of winter and nesting habitat for golden eagles in Wyoming, USA, and surrounding ecoregions. The predicted relative intensity of use from the winter-season golden eagle distribution model with relative nesting territory density from the ecoregional nesting habitat models of Dunk et al. [10]. Map shows the top 20% area for both models, with areas of overlap (green), winter only (blue), nesting only (yellow), and ecoregions (black outlines). Percent overlap and correlation within each ecoregion are shown in Table 5. Ecoregion data from the Commission for Environmental Cooperation [20] as modified by Dunk et al. [10], state boundaries from U.S. Census Bureau [21], and terrain base map modified from National Hydrography Dataset [22].

The winter and nesting models had weak to moderate positive correlations in their areas of overlap within the six ecoregions. Considering only areas of high predicted use, the top 20% of area from the models overlapped by 35–50% (Fig 7) and the top 10% of area overlapped by 23–41% (Table 5).

**Table 5:** Correlation and overlap of predicted relative intensity of use from the winter model with relative nesting territory density from the ecoregional nesting habitat models of Dunk et al. [10].

Ecoregion	Rank Correlation	Overlap of upper quantile areas (%)	
		Top 20%	Top 10%
Forested Montane	0.44	34.8	23.0
Intermontane Basins and Valleys	0.51	50.0	40.7
Northern Great Basin	0.53	45.0	31.5
Northwestern Plains	0.55	48.1	38.6
Southwestern Plains	0.29	45.9	40.5
Wyoming Basin, Uinta Basin, and North Park	0.37	40.1	32.7

Table shows the Spearman rank correlation of the models by ecoregion for the full area of overlap, and the percentage of overlap for areas in the top 20% and 10% quantile bins for each model.

### Surface management

The largest amount of predicted high-use habitat (top 10% and 20% quantiles) occurred on private lands (55%), followed by BLM (24–25%), State (8%), and U.S Forest Service (USFS; 7–8%; Table 6). All public lands combined covered 38% of the study area and contained 40% of predicted high-use areas. The amount of high-use habitat was generally proportional to the size of surface management areas, except for BLM, which had 1.56 times more high-use habitat than expected based on area, and State lands, which had 1.6 times more high-use habitat than expected.

**Table 6:** Percentage of predicted high-use winter habitat for golden eagles by surface management entity.

Surface Management Entity	Percentage of Study Area	Percentage of High-Use Habitat		Observed: Expected Ratio
		Top 20%	Top 10%	
Private	53%	55%	55%	1.04
Bureau of Land Management	16%	24%	25%	1.56
U.S. Forest Service	14%	8%	7%	0.50
Tribal	7%	4%	4%	0.57
State	5%	8%	8%	1.60
Other	2%	1%	1%	0.50
National Park Service	2%	<1%	<1%	0.17
U.S. Fish and Wildlife Service	1%	<1%	<1%	0.18

Table shows the percentage of the study area covered by each surface management entity, and the percentage of the top 20% and 10% quantile bins of predicted intensity of use occurring in each area. The observed:expected column (calculated as the ratio of the percentage of the top 10% of habitat to the percentage of the study area) shows surface management categories with more (>1) or less (<1) high-use habitat than expected based on their areal extent.

## Discussion

We developed a predictive distribution model for the wintering golden eagles in a region of the western U.S. that is critical to the conservation of the species in North America. This study fills an information gap by focusing on golden eagles not associated with the nesting habitat of territorial residents, which was historically the focus of modeling and mapping to support habitat conservation efforts [9, 10]. Specifically, our work complements available models and maps of nesting habitat by predicting relative intensity of winter use by all age classes of long-distance migrants and resident eagles not associated with nesting territories, including sub-adults and adult “floaters”, and movements of adult territory holders and their offspring outside their breeding home ranges. Similar analyses to support broad-scale spatial prioritization of raptor habitat (e.g., to reduce mortality at wind energy facilities) have been conducted in North America [9, 10] and Europe [46–48]; however, we are not aware of any that have incorporated all relevant life-history groups and seasons.

The need to account for winter use areas in conservation and management decisions for golden eagles is underscored by the relatively low degree of spatial overlap between the areas high-intensity winter use predicted by our model and high-density breeding areas from available models of nesting habitat [10]. We found that the distribution of areas with highest relative intensity of winter use by golden eagles varied among ecoregions, resulting in an uneven distribution across our study area, and corresponding opportunities to increase conservation efficiency by using our model’s predictions to target management and mitigation actions in areas where they could be expected to provide the greatest benefit.

The low degree of spatial overlap of the predicted high-use winter areas with predicted high-use breeding (Table 5 and Fig 7) strongly suggests that conservation actions focused only on local nesting habitat would not be adequate for population-level conservation of golden eagles. Within the ecoregions of the nesting models, the top 20% of area overlapped by 35–50% and the top 10% of area overlapped by only 23–41%, suggesting a conservation strategy targeting highly ranked areas for either nesting or winter would capture as much as half and as little as one quarter of high-value areas for the other season. Thus, management practices that focus on nesting habitat of resident golden eagles (e.g., stipulations for spatial-seasonal restrictions to surface development and activity around nest sites) would fail to protect a significant number of golden eagles that spend the winter in our study area. That includes long- and short-distance migrants (including breeding adults from outside our study area), resident adult and sub-adult eagles without nesting territories, and adult territory holders and their offspring outside their breeding territories. Conservation efforts that focus on the resident breeding adult segment of the population often are justified by demographic analyses that identify adult survival as the most important driver of golden eagle population persistence [49, 50]. However, long-term stability of other demographic rates is also critical [51] and our results suggest that efforts to conserve resident breeding adult eagles by targeting conservation planning, risk assessment, and mitigation strategies mainly on nesting areas would fail to cover at least half of the areas that are used by the large numbers of adult golden eagles that migrate from Alaska, Canada, and other areas to winter in our study area. Our model can be used in concert with available models of nesting habitat to identify priority areas of winter use not covered by the conservation umbrella of nesting habitat.

The concentration of wintering habitat differed among land surface management categories, suggesting that some government agencies and entities have a disproportionate responsibility for conservation of golden eagle habitat in our study region. The governmental agency with the most habitat was BLM, followed by State agencies; however, private lands had both the largest total amount of winter habitat and the most high-quality habitat (55%; Table 6). This highlights the importance of public-private collaboration in efforts to conserve winter-season habitat for golden eagles. The concentration of winter habitat on private lands also has implications for avoidance and mitigation of hazards to golden eagles, specifically from wind energy development, which has thus far occurred largely on private and non-Federal government lands within our study area, where it is not subject to the environmental review processes required for federally managed lands.

We expected relative intensity of use by golden eagles to vary regionally across our large study area and our modeling approach accounted for the influence of ecoregions. While our geographic model evaluations showed some spatial variation in model performance among subregions, overall performance was excellent among the larger ecoregions, suggesting high confidence in applying the models at that scale (Fig 4). Model performance was lowest for the Forested Montane ecoregion and more variable for the smaller subregions. Overall, grid-based evaluations showed strong concordance between predicted and observed quartiles, but that the concordance decreased with decreasing cell size. Nonetheless, the majority of prediction errors were relatively minor (one quartile), and the best predictions were in both the lowest and highest quartiles. The grid assessment provides an extra level of transparency for applying the model to management decisions and suggests that a greater degree of caution should be used for finer-scale actions (S4 Fig).

Our primary goal was to predict spatial variation in relative intensity of winter use by golden eagles, but the variables and predictions of the final model (Table 3) also provided insights into the seasonal ecology of the species in our study region. Golden eagles exhibit multi-scale selection of nesting habitat (e.g., [10, 52]) and our results suggest that winter use is also influenced by environmental conditions at a range of spatial scales (120 m to 6.4 km, Table 3). Meteorological conditions conducive to soaring flight are known to influence migratory movements of golden eagles [53] and the large contribution of wind and uplift indices to our model suggests that flight subsidies also influence habitat selection during winter. While many recent studies have focused on the influence of flight subsidies on migratory movements at fine temporal scales (e.g., [54, 55]), our results were more similar to studies of breeding habitat selection [10, 19], which have shown that golden eagles in more sedentary stages of their life-cycle also select areas with long-term average wind and orographic uplift conditions favorable to flight. Our study confirms the importance of flight subsidies as a component of winter habitat for golden eagles, building upon previous winter-season studies that inferred the importance of wind and uplift from topographic variables [56–59]. Taken together, these results suggest long-term wind and uplift conditions influence habitat selection for all life-history groups of golden eagles year-round, which has implications for siting of permanent hazards, especially wind energy projects, strings of wind turbine generators, and perhaps even individual wind turbine generators. The vegetation and land-cover variables in our model were also broadly consistent with other studies of winter-season habitat selection by golden eagles in the western U.S., which found strong positive associations with tall sagebrush and shrubland vegetation, and other variables potentially representative of prey habitat, and some evidence for avoidance of agriculture [56, 59]. By contrast, an analysis of subadult winter habitat selection in the Great Basin found higher winter use in mid-elevations, with more use of ridges, in shrub and woodland areas, closer to roads and powerlines, and at lower median wind classes [60].

Our model made accurate predictions of the relative intensity of use by golden eagles during winter among ecoregions, ecological subregions, and smaller areas (30x30- and 15x15-km grid cells), suggesting that it worked well throughout our entire study area. Our extensive evaluation process provides a high degree of confidence in the ability of the model to make accurate predictions, as well as transparency in the differences in performance that existed among geographic subregions and golden eagle life-history groups. The model’s performance with an independent GPS telemetry dataset provided further confidence in the intended application for spatial prioritization of conservation and management actions to benefit golden eagle populations.

Our single model achieved our objective to accurately predict relative intensity of winter use for all four golden eagle life-history groups included in our study (Table 4 and Fig 6). The differences in predictive performance we observed among groups were consistent with our expectations of golden eagle behavior and ecology. Adult non-migrants exhibited the strongest selection (indicated by greatest AAF ratio), which supported the hypothesis that older and more experienced individuals that reside in the study area year-round would be most selective of habitat. This aligned with evidence that adult resident golden eagles in Idaho actually increased their strength of selection during winter to target prey habitat within their expanded winter-season home ranges [61]. Strength of selection by migrant adults and non-adults was similarly moderate, which was consistent with our expectation that migrants and younger individuals not tied to breeding territories would demonstrate a lower degree of discrimination in habitat selection than resident breeders. As expected, non-adult non-migrants exhibited the lowest strength of selection. In our dataset, this life-history group comprised locations of sub-adult eagles outside their natal territories, which we expected to be the most variable, exploratory, and difficult to predict among the groups in our study [57, 62]. Nonetheless, our model was still able to rank their use nearly perfectly (Boyce Index = 0.976). Although we did not expect that our model trained on daytime use locations would also be effective at predicting use of nocturnal roosts, it still made quite accurate predictions (S5 Fig). This contrasts with examples from our study area of golden eagles leaving open habitats to roost communally in montane forests with Bald Eagles (*Haliaeetus leucocephalus* [63]). However, we expect our telemetry dataset to be more representative of the average behavior of the population, for which nocturnal roosting habitat appears to be similar overall to areas used during daytime.

### Conservation applications

Given the broad distribution and array of human-caused threats to golden eagles, our results suggest that long-term conservation of the species in North America will be most effective if it relies on proactive measures informed by spatial prioritization of high-use areas for all segments of the continental population in all seasons. The importance of a broad-scale, year-round approach to conservation for golden eagles is evident in our case study of Wyoming and surrounding ecoregions, where large numbers of migrant golden eagles augment the resident population during winter, thereby increasing the number of individuals exposed to hazards. Our winter-season distribution model provides a tool for all the parties engaged in conservation of golden eagles to identify variation in the intensity of habitat use during winter by segments of the population heretofore under-represented or ignored in conservation planning. The model is publicly available for viewing and download in a web-based mapping and decision support tool at www.raptormapper.com.

Our results suggest that successful conservation of golden eagles in our study region (regardless of migration status, age class, or breeding status) would benefit by including important winter habitat areas. The predictions from our model show that the winter-season habitat in our study area has only low-to-moderate overlap (23–50%) with nesting habitat. Moreover, areas of high-use winter habitat occur primarily in low-elevation basins and valleys that often have more human presence, private land, and development, all of which may increase the likelihood that wintering golden eagles are exposed to anthropogenic hazards such as wind turbine strikes, vehicle collisions, electrocution, and illegal shooting.

Our model can be used to maximize the efficiency of conservation efforts by targeting them in areas where they will provide the greatest benefit to golden eagles. For example, the top 10% of winter habitat value in our study area was predicted to have over 13 times greater relative intensity of use than average and 30 times greater than areas in the bottom 10% of use. Moreover, these high-use areas comprised a relatively small proportion of the landscape: the top 10% of habitat value occurred in only 0.3% (2,775 km^2^) of the study area and the top 50% of value in 1.5% (11,953 km^2^) of the area. We are not suggesting that limiting management efforts to such a small area would be sufficient to conserve wintering golden eagle populations. However, prioritization of management actions within this relatively small percentage of the landscape would be expected to provide greatly disproportionate conservation benefits (i.e., “precision conservation”).

Risk of mortality or injury to golden eagles from human-caused hazards can be conceptualized as a function of the intensity of a hazard, exposure of eagles to that hazard, and the degree of vulnerability of the individuals exposed [64, 65]. Our model predicts intensity of use by eagles, which is a measure of the exposure component of risk, but does not account for vulnerability. In some cases, pairing broad-scale models of exposure, similar to ours, with spatial data on hazards has been demonstrated to be a good predictor of overall risk (e.g., electrocution of golden eagles in the northwestern Great Plains [65]). However, exposure to a hazard may not necessarily correspond to the level of risk eagles face because their vulnerability may be influenced by other intrinsic (e.g., age, sex) and extrinsic (e.g., weather, season) factors that affect eagle behaviors such as flight altitude and foraging mode [66, 67]. Until the effectiveness of our model at predicting risk has been evaluated for specific hazards, we suggest that it be used for landscape-level conservation planning, and as an index of risk when formal risk assessments for specific threats are not available. To that end, we recommend using locations of eagle mortalities from known hazards (e.g., wind turbine strike, electrocution) to formally evaluate the ability of our model to predict risk. We developed our model to enable all parties involved in conservation and management of golden eagles to incorporate spatial prioritization of wintering habitat into decision making. Specific applications of our model results to existing decision making processes and tasks include:

1. Siting of wind energy developments as part of the “Stage 1” evaluation process described in the U.S. Fish and Wildlife Service (USFWS) Eagle Land-Based Wind Energy Guidelines, which state that “project developers should … evaluate broad geographic areas to assess the relative importance of various areas to resident breeding *and non‐breeding eagles*, and to migrant *and wintering eagles*” [emphasis added] [17]. For example, our model could be used to compare relative intensity of use by wintering eagles (exposure) among multiple potential wind energy development sites and quantify the relative risk associated with each site. Use of a consistent model framework would also facilitate review of the resulting development proposals by USFWS Office of Migratory Bird Management for compliance with the Bald and Golden Eagle Protection Act.
2. State-level planning, review, and permitting of wind energy and other developments. For example, the State of Wyoming’s Natural Resource and Energy Explorer (NREX), a web-based development pre-planning map tool, currently includes only golden eagle nesting areas [68]. Our model could be used to incorporate priority wintering areas into this and other State-level planning tools and assessments.
3. Review and consultation on a wide range of land development projects by USFWS Ecological Services Program. For example, the Wyoming Ecological Services Field Office Raptor Guidelines state that “protection of nesting, *wintering* (including communal roost sites), and foraging activities is considered essential to conserving raptors” [emphasis added] [69]. Our model could be used to incorporate relative intensity of winter use by golden eagles into project review and consultation, and to identify opportunities for eagle conservation incentives.
4. Land-use planning by Federal agencies. Our model now represents the best-available science on winter-season habitat use by golden eagles in our study area and could thus be considered in updates to land management plans (i.e., USFS Forest Plans and BLM Resource Management Plans), planning documents such as Environmental Impact Statements (EIS) and Environmental Assessments (EA) under the National Environmental Policy Act, and guidance on Best Management Practices. For example, model results could be used to compare potential impacts to eagles among alternative off-highway vehicle route locations, or in broad-scale transportation management analyses. Because our model results are publicly available, they further support the NEPA process by enabling public review of data and analyses underlying agency decisions.
5. Incorporation into other landscape prioritization initiatives and tools. Our models could represent golden eagle habitat in other spatial prioritization efforts by governmental and non-governmental organizations, for example the USFWS Sagebrush Conservation Design [70], BLM Restoration Landscapes [71], and The Nature Conservancy Wyoming Brightfields Energy Siting Initiative map tool [72].
6. Mitigation. Our model could be used to improve the efficiency of mitigation actions by targeting them in areas with greater golden eagle exposure, until such time as risk assessments are available that account for vulnerability to specific hazards. For example, mitigation of take from turbine strikes through power pole retrofits to prevent electrocution could be more efficient if implemented in areas of high eagle use, as demonstrated in Bedrosian et al. [65].
7. Proactive conservation measures. Our model could be used to target proactive conservation measures in areas with greater intensity of use by golden eagles; particularly to address hazards where risk may reasonably be assumed to be correlated with eagle density. For example, our model could be used to identify areas where golden eagles have greater exposure to vehicle strikes [15] and target management actions, like removing carcasses from roadways, or programs to reduce exposure to lead (Pb) from ammunition in gut piles of hunter-harvested big game animals, distribution of lead-free ammunition, removal of gut piles, and incentives for private landowners to require the use of lead-free ammunition.
8. Purchase of conservation easements and lands. For example, our model could be used to assess the value of potential conservation easement or land purchase areas to wintering golden eagles. Although most easements are motivated by conservation objectives for other species or values, our models could be used to quantify and compare their additive benefits to eagles. Given the scale of the model predictions and data, performance of our model is expected to be best at spatial resolutions ≥ 1 km^2^. We emphasize that the model provides an index of relative intensity of use within the extent of our study area and does not address the quality or importance of this area relative to regions outside our study area. However, we further emphasize that multiple other studies and sources of evidence suggest that our study area constitutes a continentally important area for golden eagles [11, 19]. Thus, areas where our model predicted relatively low intensity of use may still be used by golden eagles and represent relatively high-use habitat if compared to areas outside our study area.

## Supporting information

S1 Fig Modeling areas and data for analysis of golden eagle wintering distribution. Map shows training (blue) and test (red) locations in the modeled area (white) where 100,000 random background locations (not shown) were located. The model predictions were projected to the buffered study area (dark gray). State boundaries from U.S. Census Bureau [21] and terrain base map modified from National Hydrography Dataset [22].
(TIF)

S2 Fig Marginal response curves and relative contributions (% contrib.) for covariates included in a model of winter-season distribution of golden eagles in Wyoming and surrounding ecoregions. Covariates are defined in Table 3 and S1 File.
(TIF)

S3 Fig Scatterplots of predicted versus observed numbers of golden eagle winter locations among 10 equal-interval bins of relative intensity of use within 15 ecological subregions. Subregion codes are Bear Lake (BELK), Belt Mountains (BEMT), Bighorn Basin (BIBA), Central Basin and Hills (CEBH), Central High Plains (CEHP), Green River Basin (GRRB), Intermontane Basins and Valleys (IMBV), Missouri Plateau (MIPL), North Central Highlands (NOCH), Powder River Basin (PORB), Mid. and N. Rockies, Columbia and Blue Mtns., and Idaho Batholith (RCBI), Southern Rockies (SORO), Uinta Basin and North Park (UBNP), Western Great Plains North (WEGPN), Western Great Plains South (WEGPS). Subregion data from U.S. Forest Service [42] and state boundaries from U.S. Census Bureau [21].
(TIF)

S4 Fig Spatial evaluation of model predictions. Binned differences between predicted versus observed numbers of golden eagle locations in 30x30-km grid cells overlapping the modeling area for the 25% of locations (n = 8,829) withheld from the model training. Legend shows accuracy categories with matching bin ranks classified as accurately predicted and differences of 1–3 quartiles as low, medium, and high levels of over- or under-prediction. Counts of cells in each category are shown in parentheses. Table shows the proportions of quartile bin ranks of the test data within each quartile bin of predicted values. Outline of the study area and the state of Wyoming are shown as black lines. Cells with no test data are transparent. State boundaries from U.S. Census Bureau [21].
(TIF)

S5 Fig Scatterplot of predicted versus observed numbers of golden eagle winter nocturnal roost locations by life-history group among each of 10 equal-interval bins of relative intensity of use. (TIF)

S1 Table Organizations contributing golden eagle telemetry locations to the dataset used to model winter season distribution in Wyoming, USA and surrounding ecoregions. (PDF)

S1 File Candidate predictor variables. (XLSX)

S2 File Study area description and justification. (PDF)

## References

[1] Marra PP, Cohen EB, Loss SR, Rutter JE, Tonra CM. A call for full annual cycle research in animal ecology. Biology letters. 2015; 11(8):20150552. doi: 10.1098/rsbl.2015.0552 26246337 PMC4571685

[2] Pool DB, Panjabi AO, Macias-Duarte A, Solhjem DM. Rapid expansion of croplands in Chihuahua, Mexico threatens declining North American grassland bird species. Biological Conservation. 2014; 170:274–81.

[3] Fedy BC, Aldridge CL, Doherty KE, O’Donnell M, Beck JL, Bedrosian B, et al. Interseasonal movements of greater sage‐grouse, migratory behavior, and an assessment of the core regions concept in Wyoming. The Journal of Wildlife Management. 2012; 76(5):1062–71.

[4] Allen AM, Månsson J, Sand H, Malmsten J, Ericsson G, Singh NJ. Scaling up movements: from individual space use to population patterns. Ecosphere. 2016; 7(10):e01524.

[5] Katzner TE, Kochert MN, Steenhof K, McIntyre CL, Craig EH, Miller TA. Golden eagle (*Aquila chrysaetos*), version 2.0. In: Rodewald PG, Keeney BK, editors. Birds of the World. Ithaca: Cornell Lab of Ornithology; 2020. doi: 10.2173/bow.goleag.02

[6] Poessel SA, Woodbridge B, Smith BW, Murphy RK, Bedrosian BE, Bell DA, et al. Interpreting long‐distance movements of non‐migratory golden eagles: Prospecting and nomadism? Ecosphere. 2022; 13(6):e4072.

[7] Bedrosian BE, Domenech R, Shreading A, Hayes MM, Booms TL, Barger CR. Migration corridors of adult golden eagles originating in northwestern North America. PLOS ONE. 2018; 13(11):e0205204. doi: 10.1371/journal.pone.0205204 30462652 PMC6248900

[8] Eisaguirre JM, Booms TL, Barger CP, Lewis SB, Breed GA. Novel step selection analyses on energy landscapes reveal how linear features alter migrations of soaring birds. Journal of Animal Ecology. 2020; 89(11):2567–83. doi: 10.1111/1365-2656.13335 32926415

[9] Tack JD, Fedy BC. Landscapes for energy and wildlife: conservation prioritization for golden eagles across large spatial scales. PLOS ONE. 2015; 10(8):e0134781. doi: 10.1371/journal.pone.0134781 26262876 PMC4532434

[10] Dunk JR, Woodbridge B, Lickfett TM, Bedrosian G, Noon BR, LaPlante DW, et al. Modeling spatial variation in density of golden eagle nest sites in the western United States. PLOS ONE. 2019; 14(9):e0223143. doi: 10.1371/journal.pone.0223143 31568505 PMC6768475

[11] Wallace Z, Bedrosian G, Woodbridge B, Williams G, Bedrosian BE, Dunk J. Wyoming and Uinta Basins golden eagle conservation strategy. Denver: U.S. Fish and Wildlife Service Western Golden Eagle Team; 2019. Available: https://ecos.fws.gov/ServCat/DownloadFile/167909.

[12] Bedrosian BE, Wallace Z, Bedrosian G., Woodbridge B, Dunk J. Northwestern Plains golden eagle conservation strategy. Denver: U.S. Fish and Wildlife Service Western Golden Eagle Team; 2019. Available: https://ecos.fws.gov/ServCat/Reference/Profile/98141.

[13] Bedrosian B, Craighead D, Crandall R. Lead exposure in bald eagles from big game hunting, the continental implications and successful mitigation efforts. PLOS ONE. 2012; 7(12):e51978. doi: 10.1371/journal.pone.0051978 23284837 PMC3526477

[14] Lonsdorf E, Sanders‐Reed CA, Boal C, Allison TD. Modeling golden eagle‐vehicle collisions to design mitigation strategies. The Journal of Wildlife Management. 2018; 82(8):1633–44.

[15] Slater SJ, Maloney DM, Taylor JM. Golden eagle use of winter roadkill and response to vehicles in the western United States. The Journal of Wildlife Management. 2022; 86(6):e22246.

[16] U.S. Fish and Wildlife Service. National bald eagle management guidelines. Washington, DC: U.S. Fish and Wildlife Service; 2017.

[17] U.S. Fish and Wildlife Service. Eagle conservation plan guidance, module 1–land-based wind energy, version 2. Washington, DC: Division of Migratory Bird Management; 2013.

[18] Nielson RM, DiDonato GT, McDonald LL. A survey of golden eagles (*Aquila chrysaetos*) in the Western U.S. mid-winter 2017. Cheyenne: Western EcoSystems Technology, Inc.; 2017. Available: https://irma.nps.gov/DataStore/DownloadFile/581855.

[19] Nielson RM, Murphy RK, Millsap BA, Howe WH, Gardner G. Modeling late-summer distribution of golden eagles (*Aquila chrysaetos*) in the western United States. PLOS ONE. 2016; 11(8):e0159271. 27556735 10.1371/journal.pone.0159271 PMC4996490

[20] Wiken ED, Nava FJ, Griffith G. North American terrestrial ecoregions—level III. Montreal: Commission for Environmental Cooperation; 2011. Available: http://www.cec.org/files/documents/publications/10415-north-american-terrestrial-ecoregionslevel-iii-en.pdf

[21] U.S. Census Bureau. 2018 [Accessed 1 August 2022]. Cartographic Boundary Files. Available: https://www.census.gov/geographies/mapping-files/time-series/geo/carto-boundary-file.html.

[22] National Hydrography Dataset Plus [raster and vector digital data set]. 2012. Edition 2.10. Washington, D.C.: U.S. Environmental Protection Agency. Available: http://www.horizon-systems.com/NHDPlus/index.php.

[23] Collecte Localisation Satellites. Argos User’s Manual. Toulouse: Collecte Localisation Satellites. 2011. Available: http://www.argos-system.org/html/userarea/manual_en.html.

[24] McIntyre CL, Douglas DC, Collopy MW. Movements of golden eagles (*Aquila chrysaetos*) from interior Alaska during their first year of independence. The Auk. 2008; 125(1):214–24.

[25] Douglas D. The Douglas Argos-filter Program. 2012. Available: https://www.usgs.gov/media/files/douglas-argos-filter-algorithm.

[26] National Oceanic and Atmospheric Administration. 2022 [cited 2 January 2022]. NOAA Solar Calculator, Solar Calculation Details. Available: https://gml.noaa.gov/grad/solcalc/calcdetails.html.

[27] Watson JW, Duff AA, Davies RW. Home range and resource selection by GPS‐monitored adult golden eagles in the Columbia Plateau Ecoregion: implications for wind power development. The Journal of Wildlife Management. 2014; 78(6):1012–21.

[28] Worton BJ. Kernel methods for estimating the utilization distribution in home‐range studies. Ecology. 1989, 70:164–168.

[29] Crandall RH, Bedrosian BE, Craighead D. Habitat selection and factors influencing nest survival of golden eagles in south-central Montana. Journal of Raptor Research. 2015; 49(4):413–28.

[30] Torres LG, Orben RA, Tolkova I, Thompson DR. Classification of animal movement behavior through residence in space and time. PLOS ONE. 2017 Jan 3;12(1):e0168513. doi: 10.1371/journal.pone.0168513 28045906 PMC5207689

[31] Tredennick AT, Hooker G, Ellner SP, Adler PB. A practical guide to selecting models for exploration, inference, and prediction in ecology. Ecology. 2021; 102(6):e03336. doi: 10.1002/ecy.3336 33710619 PMC8187274

[32] Phillips SJ, Anderson RP, Schapire RE. Maximum entropy modeling of species geographic distributions. Ecological modelling. 2006; 190(3–4):231–59.

[33] Merow C, Smith MJ, Silander JA Jr. A practical guide to MaxEnt for modeling species’ distributions: what it does, and why inputs and settings matter. Ecography. 2013; 36(10):1058–69.

[34] Nielson RM, Sawyer H. Estimating resource selection with count data. Ecology and evolution. 2013; 3(7):2233–40. doi: 10.1002/ece3.617 23919165 PMC3728960

[35] Guisan A, Zimmermann NE. Predictive habitat distribution models in ecology. Ecological modelling. 2000; 135(2–3):147–86.

[36] Elith J, Leathwick JR. Species distribution models: ecological explanation and prediction across space and time. Annual review of ecology, evolution, and systematics. 2009; 40:677–97.

[37] Yates KL, Bouchet PJ, Caley MJ, Mengersen K, Randin CF, Parnell S, et al. Outstanding challenges in the transferability of ecological models. Trends in Ecology & Evolution. 2018; 33(10):790–802. doi: 10.1016/j.tree.2018.08.001 30166069

[38] Phillips SJ. 2017 [cited 3 March 2023]. A brief tutorial on Maxent. Available: https://biodiversityinformatics.amnh.org/open_source/maxent/Maxent_tutorial_2021.pdf.

[39] Boyce MS, Vernier PR, Nielsen SE, Schmiegelow FK. Evaluating resource selection functions. Ecological modelling. 2002; 157(2–3):281–300.

[40] Hirzel AH, Le Lay G, Helfer V, Randin C, Guisan A. Evaluating the ability of habitat suitability models to predict species presences. Ecological modelling. 2006; 199(2):142–52.

[41] Roberts DR, Bahn V, Ciuti S, Boyce MS, Elith J, Guillera‐Arroita G, et al. Cross‐validation strategies for data with temporal, spatial, hierarchical, or phylogenetic structure. Ecography. 2017; 40(8):913–29.

[42] Cleland DT, Freeouf JA, Keys JE Jr., Nowacki GJ, Carpenter C, McNab WH. Ecological sub-regions: sections and subsections of the conterminous United States [1:3,500,000] [CD-ROM]. Sloan, A.M., cartog. Gen. Tech. Report WO-76. Washington, DC: Department of Agriculture, Forest Service; 2007.

[43] Dunk JR, Woodbridge B, Schumaker N, Glenn EM, White B, LaPlante DW, et al. Conservation planning for species recovery under the Endangered Species Act: A case study with the northern spotted owl. PLOS ONE. 2019; 14(1):e0210643. doi: 10.1371/journal.pone.0210643 30640947 PMC6331132

[44] Dunk JR, Hawley JJ. Red-tree vole habitat suitability modeling: implications for conservation and management. Forest Ecology and Management. 2009; 258(5):626–34.

[45] Zielinski WJ, Dunk JR, Yaeger JS, LaPlante DW. Developing and testing a landscape-scale habitat suitability model for fisher (*Martes pennanti*) in forests of interior northern California. Forest Ecology and Management. 2010; 260(9):1579–91.

[46] Tikkanen H, Rytkönen S, Karlin OP, Ollila T, Pakanen VM, Tuohimaa H, et al. Modelling golden eagle habitat selection and flight activity in their home ranges for safer wind farm planning. Environmental Impact Assessment Review. 2018; 71:120–31.

[47] Vignali S, Lörcher F, Hegglin D, Arlettaz R, Braunisch V. A predictive flight-altitude model for avoiding future conflicts between an emblematic raptor and wind energy development in the Swiss Alps. Royal Society Open Science. 2022; 9(2):211041. doi: 10.1098/rsos.211041 35154790 PMC8826134

[48] Tikkanen H, Balotari-Chiebao F, Laaksonen T, Pakanen VM, Rytkönen S. Habitat use of flying subadult white-tailed eagles (*Haliaeetus albicilla*): implications for land use and wind power plant planning. Ornis Fennica. 2018; 95(4).

[49] Tack JD, Noon BR, Bowen ZH, Strybos L, Fedy BC. No substitute for survival: perturbation analyses using a golden eagle population model reveal limits to managing for take. Journal of Raptor Research. 2017; 51(3):258–72.

[50] Millsap BA, Zimmerman GS, Kendall WL, Barnes JG, Braham MA, Bedrosian BE, et al. Age‐specific survival rates, causes of death, and allowable take of golden eagles in the western United States. Ecological Applications. 2022; 32(3):e2544. doi: 10.1002/eap.2544 35080801 PMC9286660

[51] Katzner T, Milner‐Gulland EJ, Bragin E. Using modeling to improve monitoring of structured populations: are we collecting the right data? Conservation Biology. 2007; 21(1):241–52. doi: 10.1111/j.1523-1739.2006.00561.x 17298530

[52] Squires JR, Olson LE, Wallace ZP, Oakleaf RJ, Kennedy PL. Resource selection of apex raptors: implications for siting energy development in sagebrush and prairie ecosystems. Ecosphere. 2020; 11(8):e03204.

[53] Duerr AE, Miller TA, Lanzone M, Brandes D, Cooper J, O’Malley K, et al. Flight response of slope‐soaring birds to seasonal variation in thermal generation. Functional Ecology. 2015; 29(6):779–90.

[54] Eisaguirre JM, Auger-Méthé M, Barger CP, Lewis SB, Booms TL, Breed GA. Dynamic-parameter movement models reveal drivers of migratory pace in a soaring bird. Frontiers in Ecology and Evolution. 2019; 7:317.

[55] Sandhu R, Tripp C, Quon E, Thedin R, Lawson M, Brandes D, et al. Stochastic agent-based model for predicting turbine-scale raptor movements during updraft-subsidized directional flights. Ecological Modelling. 2022; 466:109876.

[56] Domenech R, Bedrosian BE, Crandall RH, Slabe VA. Space use and habitat selection by adult migrant golden eagles wintering in the western United States. Journal of Raptor Research. 2015; 49(4):429–40.

[57] Poessel SA, Bloom PH, Braham MA, Katzner TE. Age-and season-specific variation in local and long-distance movement behavior of golden eagles. European Journal of Wildlife Research. 2016; 62:377–93.

[58] Miller TA, Brooks RP, Lanzone MJ, Cooper J, O’Malley K, Brandes D, et al. Summer and winter space use and home range characteristics of golden eagles (*Aquila chrysaetos*) in eastern North America. The Condor: Ornithological Applications. 2017; 119(4):697–719.

[59] Craig EH, Fuller MR, Craig TH, Huettmann F. Assessment of potential risks from renewable energy development and other anthropogenic factors to wintering golden eagles in the western United States. In: Humphries G, Magness DR, Huettmann F, editors. Machine Learning for Ecology and Sustainable Natural Resource Management. Zurich: Springer; 2018. pp. 379–407.

[60] Hixson KM, Slater SJ, Knight RN, Lonsinger RC. Seasonal variation in resource selection by subadult golden eagles in the Great Basin Desert. Wildlife Biology. 2022; 2022(1):e01002.

[61] Marzluff JM, Knick ST, Vekasy MS, Schueck LS, Zarriello TJ. Spatial use and habitat selection of golden eagles in southwestern Idaho. The Auk. 1997; 114(4):673–87.

[62] Murphy RK, Dunk JR, Woodbridge B, Stahlecker DW, LaPlante DW, Millsap BA, et al. First-year dispersal of golden eagles from natal areas in the southwestern United States and implications for second-year settling. Journal of Raptor Research. 2017; 51(3):216–33.

[63] Bagdonas K, Snyder L, Rodeman K, Milliken G, Blau G. A study of the effect of drilling on eagle behavior at overwintering roost sites on Pine Mountain, Natrona County, Wyoming: Final Report to Moncrief Oil. University of Wyoming, Research and Statistical Consulting Center; 1985.

[64] Connelly A, Carter J, Handley J, Hincks S. Enhancing the practical utility of risk assessments in climate change adaptation. Sustainability. 2018; 10(5):1399.

[65] Bedrosian G, Carlisle JD, Woodbridge B, Dunk JR, Wallace ZP, Dwyer JF, et al. A spatially explicit model to predict the relative risk of golden eagle electrocutions in the Northwestern Plains, USA. Journal of Raptor Research. 2020; 54(2):110–25.

[66] Hunt G. Golden eagles in a perilous landscape: predicting the effects of mitigation for wind turbine blade-strike mortality. California Energy Commission. 2002.

[67] Mojica EK, Dwyer JF, Harness RE, Williams GE, Woodbridge B. Review and synthesis of research investigating golden eagle electrocutions. The Journal of Wildlife Management. 2018; 82(3):495–506.

[68] State of Wyoming. 2022 [cited 30 December 2022]. Natural Resource and Energy Explorer (NREX): a web GIS-based tool that supports pre-planning development considerations. Available: https://nrex.wyo.gov/.

[69] U.S. Fish and Wildlife Service. 2022 [Accessed 15 August 2022]. Wyoming Ecological Services Field Office Raptor Guidelines. Available: https://www.fws.gov/sites/default/files/documents/wyoming-ecological-services-field-office-raptor-guidelines-2022-03-09.pdf.

[70] Doherty K, Theobald DM, Bradford JB, Wiechman LA, Bedrosian G, Boyd CS, et al. A sagebrush conservation design to proactively restore America’s sagebrush biome: U.S. Geological Survey Open-File Report 2022–1081. 2022, 38 p. Available: 10.3133/ofr20221081.

[71] Bureau of Land Management. 2023 [Accessed December 11, 2023]. Restoration Landscapes. Available: https://storymaps.arcgis.com/stories/6966af5d6f584f8b80f102d391671a3f.

[72] The Nature Conservancy. 2023 [Accessed December 11, 2023]. Wyoming brightfields energy siting initiative map tool. Available: https://tnc.maps.arcgis.com/apps/webappviewer/index.html?id=1cf531c47ab841db9dc93614f1a6cdf3.

