# Supplementary figures and images for: Predicting the spatial distribution of wintering golden eagles to inform full annual cycle conservation in western North America
Source: PLoS One. 2024 Jan 31;19(1):e0297345. doi: 10.1371/journal.pone.0297345 (PMC10830038; doi:10.1371/journal.pone.0297345)

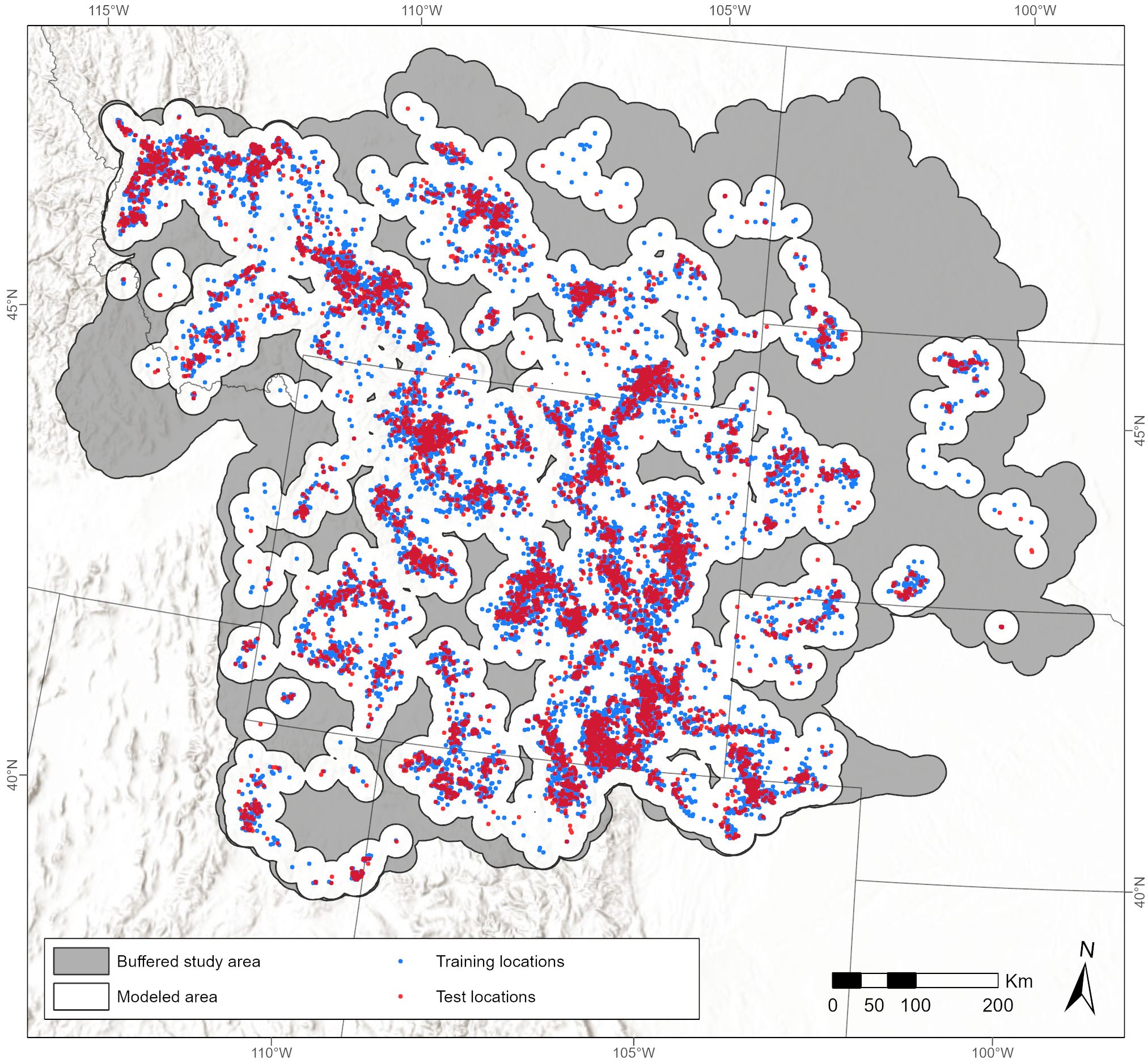

Supplement: S1 Fig — Map shows training (blue) and test (red) locations in the modeled area (white) where 100,000 random background locations (not shown) were located. The model predictions were projected to the buffered study area (dark gray). State boundaries from U.S. Census Bureau [21] and terrain base map modified from National Hydrography Dataset [22]. (TIF) [file pone.0297345.s001.tif]

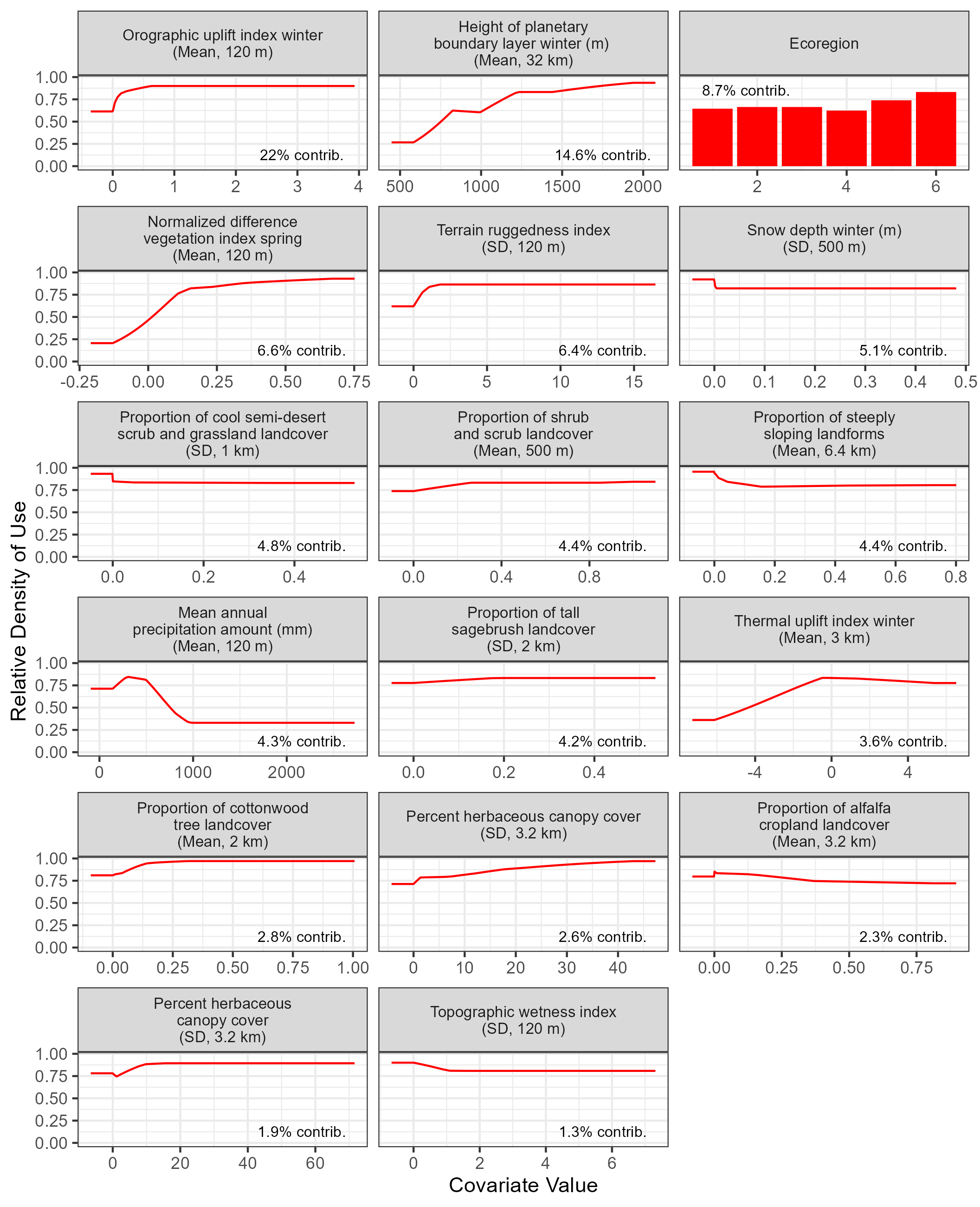

Supplement: S2 Fig — Covariates are defined in Table 3 and S1 File. (TIF) [file pone.0297345.s002.tif]

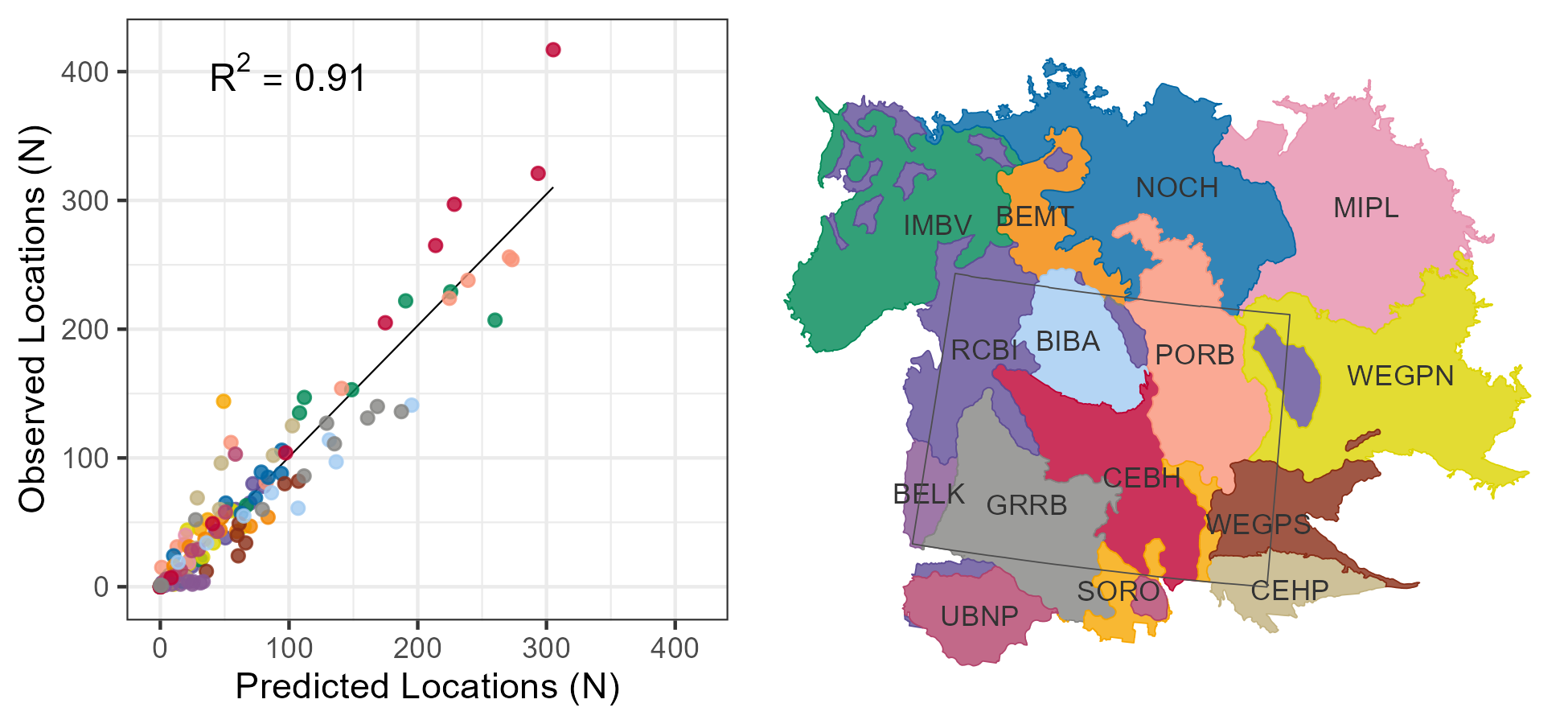

Supplement: S3 Fig — Subregion codes are Bear Lake (BELK), Belt Mountains (BEMT), Bighorn Basin (BIBA), Central Basin and Hills (CEBH), Central High Plains (CEHP), Green River Basin (GRRB), Intermontane Basins and Valleys (IMBV), Missouri Plateau (MIPL), North Central Highlands (NOCH), Powder River Basin (PORB), Mid. and N. Rockies, Columbia and Blue Mtns., and Idaho Batholith (RCBI), Southern Rockies (SORO), Uinta Basin and North Park (UBNP), Western Great Plains North (WEGPN), Western Great Plains South (WEGPS). Subregion data from U.S. Forest Service [42] and state boundaries from U.S. Census Bureau [21]. (TIF) [file pone.0297345.s003.tif]

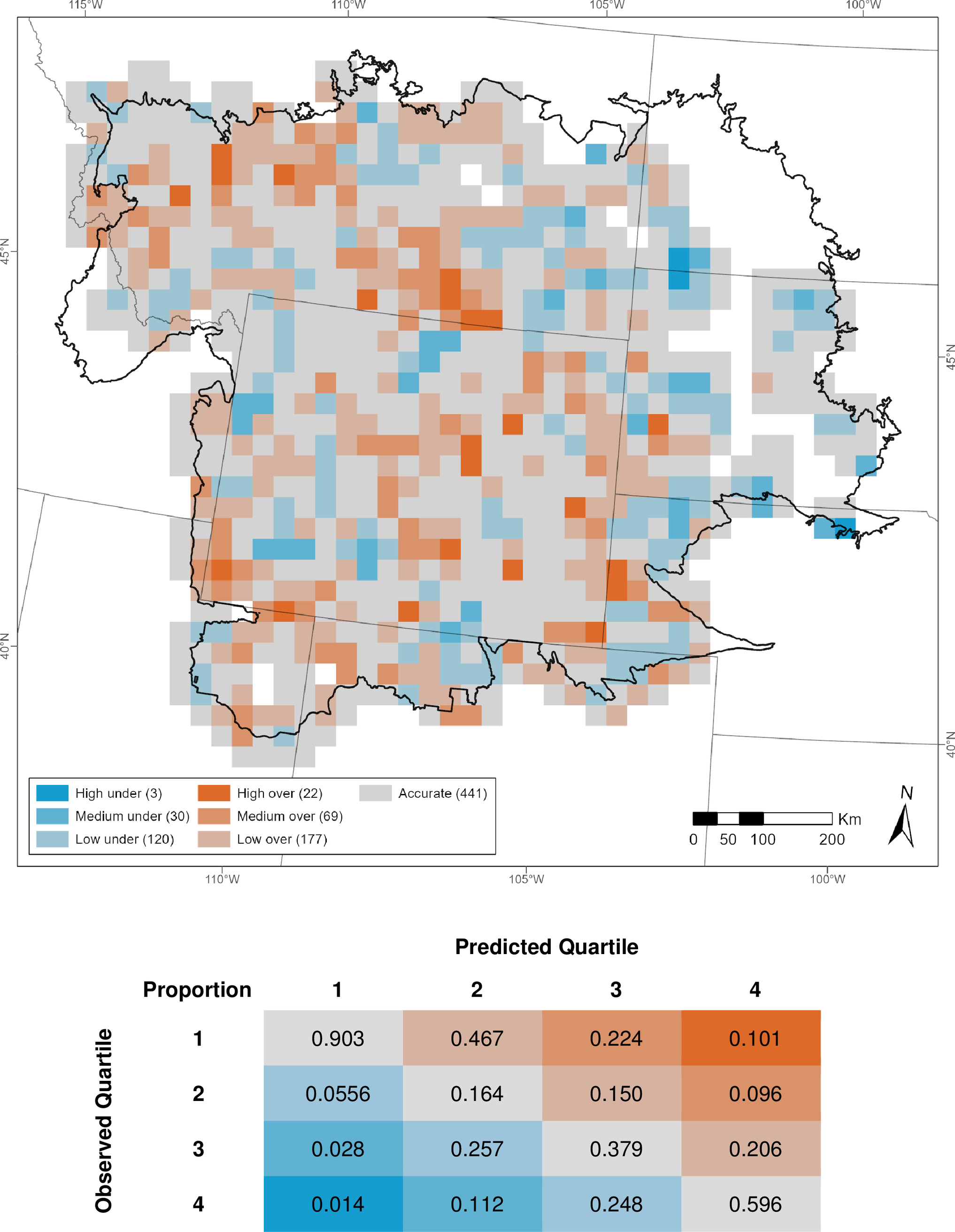

Supplement: S4 Fig — Binned differences between predicted versus observed numbers of golden eagle locations in 30x30-km grid cells overlapping the modeling area for the 25% of locations (n = 8,829) withheld from the model training. Legend shows accuracy categories with matching bin ranks classified as accurately predicted and differences of 1–3 quartiles as low, medium, and high levels of over- or under-prediction. Counts of cells in each category are shown in parentheses. Table shows the proportions of quartile bin ranks of the test data within each quartile bin of predicted values. Outline of the study area and the state of Wyoming are shown as black lines. Cells with no test data are transparent. State boundaries from U.S. Census Bureau [21]. (TIF) [file pone.0297345.s004.tif]

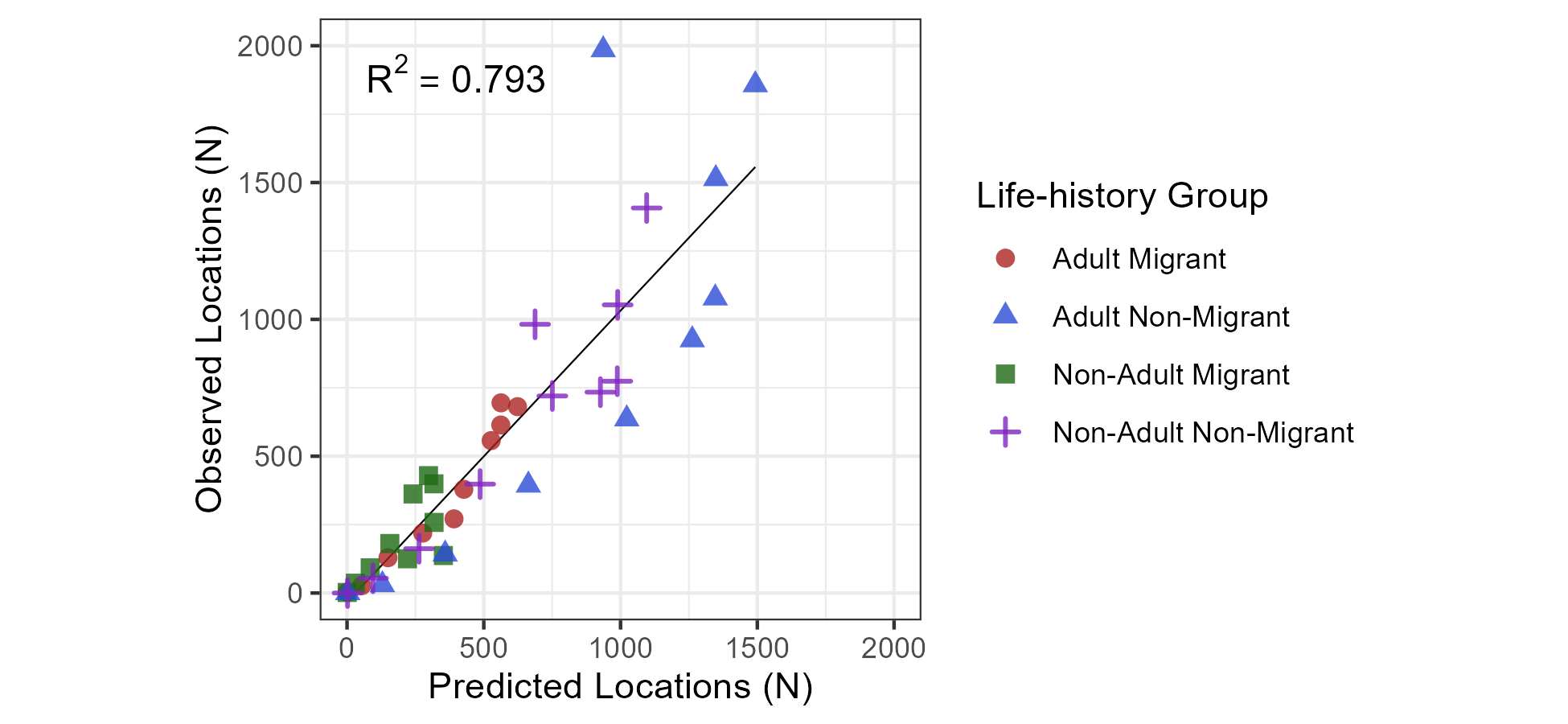

Supplement: S5 Fig — (TIF) [file pone.0297345.s005.tif]
