# Supplementary material for: Predicting the spatial distribution of wintering golden eagles to inform full annual cycle conservation in western North America
Source: PLoS One. 2024 Jan 31;19(1):e0297345. doi: 10.1371/journal.pone.0297345 (PMC10830038; doi:10.1371/journal.pone.0297345)
Supplement: S1 Table — (PDF) [file pone.0297345.s006.pdf]

S1 Table. Organizations contributing golden eagle telemetry locations to the dataset used to model winter season distribution in Wyoming, USA and surrounding ecoregions.

Organization	Data points (% of total)
U.S. Fish and Wildlife Service, Region 6	38
Teton Raptor Center	19
Raptor View Research Institute	10
U.S. Geological Survey, Conservation Science Global, Wildlands Bio-consulting	9*
Craighead Beringia South	8
U.S. Geological Survey Alaska	7
Wildlife Research Institute	4
Alaska Department of Fish and Game	2
HawkWatch International	2
USFWS National Raptor Team	<1

* Independent test data only
