# Supplementary material for: Predicting the spatial distribution of wintering golden eagles to inform full annual cycle conservation in western North America
Source: PLoS One. 2024 Jan 31;19(1):e0297345. doi: 10.1371/journal.pone.0297345 (PMC10830038; doi:10.1371/journal.pone.0297345)
Supplement: S2 File — (PDF) [file pone.0297345.s008.pdf]

S1 Text. Study area description and justification.

Our 765,953-km^2 study area was based on the golden eagle modeling ecoregions of Dunk et al. [1] that overlapped the state of Wyoming, USA. We excluded portions of some ecoregions outside Wyoming where we judged that golden eagle habitat differed substantially from their area within the state. In response to feedback from land managers, we made minor modifications to ecoregion boundaries to fully cover administrative units (e.g., U.S. Bureau of Land Management (BLM) Field Offices, and U.S. Forest Service (USFS) Regions) where such changes did not result in the inclusion of large areas of dissimilar habitat. The modeling regions of Dunk et al. (2019) were based on the level-III ecoregions defined by the Commission on Environmental Cooperation (CEC) [2]. Our study area included all of the CEC Middle Rockies, Wyoming Basin, and Northwestern Great Plains ecoregions, which together defined its western, northern, and northeastern boundaries. The southern boundary was defined by a portion of the Southern Rockies ecoregion modified slightly to align with the boundaries of the Vernal and Little Snake Bureau of Land Management Field Offices, and portions of the Wasatch and Uinta Mountains and Colorado Plateaus ecoregions that Dunk et al. [1] included in the Uinta Basin ecoregion because of their similarity to the Southern Rockies and Wyoming Basin ecoregions, respectively. The southeastern boundary was defined by the High Plains ecoregion north of the South Platte River, which was similar to the extent of that ecoregion in Wyoming due to relatively low density of tilled agriculture and urban development. Finally, we included the Intermontane Basins and Valleys ecoregion, even though it did not overlap Wyoming, because it was interspersed with the Forested Montane and Northwestern Plains ecoregions in Montana, where it had similar habitat to other portions of the study area.

References:

1. Dunk, J.R., Woodbridge, B., Lickfett, T.M., Bedrosian, G., Noon, B.R., LaPlante, D.W., Brown, J.L. and Tack, J.D., 2019. Modeling spatial variation in density of golden eagle nest sites in the western United States. *PLOS ONE*, *14*(9), p.e0223143.
2. Wiken, E., Nava, F.J. and Griffith, G., 2011. North American terrestrial ecoregions—level III. Commission for Environmental Cooperation, Montreal, Quebec, Canada.
